# *Artemisia* spp.: An Update on Its Chemical Composition, Pharmacological and Toxicological Profiles

**DOI:** 10.1155/2022/5628601

**Published:** 2022-09-05

**Authors:** Javad Sharifi-Rad, Jesús Herrera-Bravo, Prabhakar Semwal, Sakshi Painuli, Himani Badoni, Shahira M. Ezzat, Mai M. Farid, Rana M. Merghany, Nora M. Aborehab, Mohamed A. Salem, Surjit Sen, Krishnendu Acharya, Natallia Lapava, Miquel Martorell, Bekzat Tynybekov, Daniela Calina, William C. Cho

**Affiliations:** ^1^Facultad de Medicina, Universidad del Azuay, Cuenca, Ecuador; ^2^Departamento de Ciencias Básicas, Facultad de Ciencias, Universidad Santo Tomas, Chile; ^3^Center of Molecular Biology and Pharmacogenetics, Scientific and Technological Bioresource Nucleus, Universidad de La Frontera, Temuco 4811230, Chile; ^4^Department of Life Sciences, Graphic Era Deemed To Be University, Dehradun, 248002, Uttarakhand, India; ^5^Uttarakhand Council for Biotechnology (UCB), Prem Nagar, Dehradun, 248007 Uttarakhand, India; ^6^Department of Biotechnology, School of Applied and Life Sciences, Uttaranchal University, Prem Nagar, Dehradun, 248007, Uttarakhand, India; ^7^Department of Pharmacognosy, Faculty of Pharmacy, Cairo University, Cairo 11562, Egypt; ^8^Department of Pharmacognosy, Faculty of Pharmacy, October University for Modern Sciences and Arts (MSA), Giza 12451, Egypt; ^9^Department of Phytochemistry and Plant Systematics, National Research Centre, 33 El Bohouth St., Dokki, P. O. 12622, Giza, Egypt; ^10^Pharmacognosy Department, Pharmaceutical and Drug Industries Research Institute, National Research Centre (NRC), 33 El-Bohouth street, Dokki, Giza, Egypt; ^11^Department of Biochemistry, Faculty of Pharmacy, October University for Modern Sciences and Arts (MSA), Giza 12451, Egypt; ^12^Department of Pharmacognosy, Faculty of Pharmacy, Menoufia University, Gamal Abd El Nasr St., Shibin El Kom, 32511 Menoufia, Egypt; ^13^Molecular and Applied Mycology and Plant Pathology Laboratory, Department of Botany, University of Calcutta, Kolkata 700019, India; ^14^Department of Botany, Fakir Chand College, Diamond Harbour, West Bengal 743331, India; ^15^Medicine Standardization Department, Vitebsk State Medical University, Belarus; ^16^Department of Nutrition and Dietetics, Faculty of Pharmacy, And Centre for Healthy Living, University of Concepción, Concepción, Chile; ^17^Universidad de Concepción, Unidad de Desarrollo Tecnológico (UDT), 4070386 Concepción, Chile; ^18^Department of Biodiversity of Bioresources, Al-Farabi Kazakh National University, Almaty, Kazakhstan; ^19^Department of Clinical Pharmacy, University of Medicine and Pharmacy of Craiova, 200349 Craiova, Romania; ^20^Department of Clinical Oncology, Queen Elizabeth Hospital, Kowloon, Hong Kong

## Abstract

*Artemisia* plants are traditional and ethnopharmacologically used to treat several diseases and in addition in food, spices, and beverages. The genus is widely distributed in all continents except the Antarctica, and traditional medicine has been used as antimalarial, antioxidant, anticancer, antinociceptive, anti-inflammatory, and antiviral agents. This review is aimed at systematizing scientific data on the geographical distribution, chemical composition, and pharmacological and toxicological profiles of the *Artemisia* genus. Data from the literature on *Artemisia* plants were taken using electronic databases such as PubMed/MEDLINE, Scopus, and Web of Science. Selected papers for this updated study included data about phytochemicals, preclinical pharmacological experimental studies with molecular mechanisms included, clinical studies, and toxicological and safety data. In addition, ancient texts and books were consulted. The essential oils and phytochemicals of the *Artemisia* genus have reported important biological activities, among them the artemisinin, a sesquiterpene lactone, with antimalarial activity. *Artemisia absinthium* L. is one of the most famous *Artemisia* spp. due to its use in the production of the absinthe drink which is restricted in most countries because of neurotoxicity. The analyzed studies confirmed that *Artemisia* plants have many traditional and pharmacological applications. However, scientific data are limited to clinical and toxicological research. Therefore, further research is needed on these aspects to understand the full therapeutic potential and molecular pharmacological mechanisms of this medicinal species.

## 1. Introduction

The search and development of medicines from plant raw materials have been one of the important areas of human science for centuries [1]. A new breath of this scientific direction was given by the discovery of unique antimalarial agent artemisinin by the Chinese scientist Youyou Tu, for which she received the Nobel Prize in Physiology or Medicine in 2015. The source of this medicine was the *Artemisia annua* L., which has long been known in Chinese folk medicine [2]. This plant is not the only one of the well-known representatives of the *Artemisia* L. genus. *Artemisia* genus (*Asteraceae*), named after the Greek goddess of the hunt and fertility Artemis, is considered one of the most widely distributed genera all over the world [3] and unites more than 400 species of plants of various life forms (grasses, shrubs, and less often trees) [4]. Many of them are weeds, some are invasive in certain regions of the planet, and at the same time, some species are listed in the Red Book. Wide distribution, a variety of component composition, and the resulting wide range of pharmacological effects made plants of the *Artemisia* genus popular remedies of traditional medicine and ensured their study and subsequent introduction into official medicine [5–7].

The medicinal and aromatic applications of *Artemisia* are well known for a long time as it produces volatile oil which has applications in medicine, cosmetics, and food production [8]. Some species of *Artemisia* are edible [9], and others especially those grown in Korea have been traditionally applied in treating inflammations and ulcers. The most famous species around the world are *A. annua* and *A. absinthium* L. which are known for their uses in traditional medicine [10, 11]. In South Africa, *Artemisia afra* Jacq. ex Willd. is commonly used for treating inflammation, coughs, colds, malaria, fever, influenza, and diabetes [12], while, in North America, *Artemisia dracunculus* L. is widely used for the treatment of wounds and possesses antioxidant and antidiabetic activities [13, 14]. Furthermore, *Artemisia vulgaris* L. is used in traditional medicine and has several activities such as anticancer, hepatoprotective, antiepileptic, antimalarial, and insecticidal properties [15–19]. Other various species such as *Artemisia nilagirica* (C.B.Clarke) Pamp., *A. dracunculus*, *Artemisia herba-alba* Asso, *A. armeniaca* Lam., and *Artemisia scoparia* Waldst. & Kitam. possess significant therapeutic properties [20]. *A. scoparia* also has a long history in medicine, and it has been in clinics as a diuretic, choleretic, and hepatoprotective [21]. *A. scoparia* was used to treat hepatitis, jaundice, sores, pruritus, asthma, gastritis, and expel parasites and to treat spiders' bites. A combination of *A. scoparia*, *Gardenia jasminoides* J. Ellis, and rhubarb (*Rheum rhabarbarum* L.) was reported to be a classical prescription for curing jaundice [21]. Currently, several biopharmaceutical products containing *Artemisia* extracts are available nowadays in the local markets to treat various diseases [22]. The purpose of the review is to systematize updated scientific data on the chemical composition and new insights in the pharmacological mechanisms of action and discusses the toxicological profiles of the *Artemisia* genus.

## 2. Methodology

Two biomedical literature databases were searched for this review: PubMed/MEDLINE and Web of Science using the following MeSH search terms: “Artemisia/chemistry,” “Phytochemicals,” “Artemisinins/pharmacology,, “Artemisinins/therapeutic use,” “Medicine, Traditional,” “Phytotherapy/methods,” “Plant Extracts/pharmacology,” “Plant Extracts/therapeutic use,” “Plant Oils /pharmacology,” “Plant Oils/therapeutic use,” “Structure-Activity Relationship,” “Animals,” and “Humans.” Inclusion criteria are as follows: preclinical and clinical studies on the sources, acquisition approaches, experimental pharmacology, toxicology, and safety data regarding *Artemisia* spp. were included. Both in vivo and in vitro pharmacological studies which underlie the molecular mechanism of action were included. Exclusion criteria are as follows: studies with data not relevant for the aim of this updated review, or poor quality of studies, duplicate studies. The taxonomy of plant species has been validated using the World Flora Online [23].

## 3. Geographical Distribution of *Artemisia* spp.

**Figure 1:**
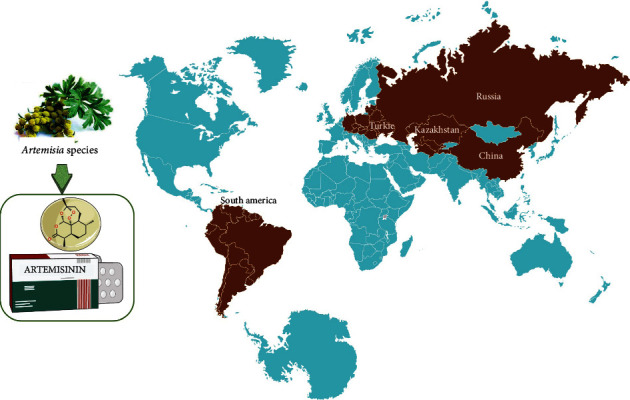
Geographical distribution of *Artemisia* species.

The genus *Artemisia* is widely cosmopolitan and distributed worldwide except the Antarctica [24–26]. The genus is heterogeneous and inhabits from the sea level to high altitudes of around 4000 masl (meters above *sea level*) [27]. The species of *Artemisia* grows abundant in the Northern Hemisphere, and a low degree of colonization has been reported in the Southern Hemisphere [27, 28]. The main centre of species diversity of *Artemisia* is located in Central Asia consisting the region of Uzbekistan, Tadzhikistan, Turkmenistan, Kazakhstan, Kyrgyzstan, parts of Russia, China, and Mongolia. Other relevant centres of diversity include the territory of Iran-Turanian and Mediterranean regions and in western North America [29–33]. *Artemisia* has been spread beyond its native origin and successfully distributed and colonized in most of the arctic-alpine, temperate, and subtropical zones of the Northern Hemisphere. The distribution of the genus from Northern Asia primarily follows the main three routes: (1) in the West, it migrates into Europe, Western Asia, Mediterranean Basin, and Africa; (2) Siberia and into western North America; and (3) further south into Asia [34, 35]. Only a few number of species, not exceeding 25 taxa, have been reported from the Southern Hemisphere although a small diversity centre occurs in South America and it is found in Oceania as allochthonous taxa [32] (Figure 1).

## 4. Phytochemical Composition

### 4.1. Essential Oils

The essential oils (EOs) present in botanicals have been used for centuries in the form of spices, medicines, and their pleasant odour [36]. It has been possible only due to the development of distillation techniques in the middle ages and is used in their ancient applications in food, drugs, or cosmetics [37]. While, in the last decades, the EO industry entered different sectors with new dimensions and targets due to its various therapeutic applications. The chemical composition of *Artemisia* genus EOs has been reported by several authors around the world. The composition of EOs varies depending on several factors including the plant part, growing season, age of the plant, location, extraction techniques, solvent, and timing [38]. The detailed investigations on the EO composition of the *Artemisia* genus from different geographical regions have been presented in Table 1.

### 4.2. Other Bioactive Compounds

The phytochemical diversity assessment of the *Artemisia* genus exhibited the presence of different types of secondary metabolites reported by several authors around the world (Table 2).

## 5. Pharmacological Effects of *Artemisia* spp. Extracts and Its Bioactives: Underlying Molecular Mechanisms

**Figure 2:**
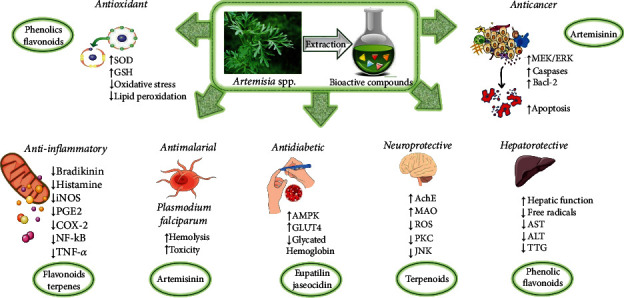
Illustrative scheme with the most representative pharmacological properties of bioactives of *Artemisia* spp. and their potential mechanisms of action. ↑: increase; ↓: decrease; SOD: superoxide dismutase; GSH: glutathione; MEK/ERK: mitogen-activated extracellular signal-regulated kinase/extracellular signal-regulated protein kinase; Bcl-2: B-cell lymphoma 2; *iNOS*: inducible *nitric oxide* synthase; PGE2: prostaglandin E2; *COX*-*2*: cyclooxygenase-2; NF-*κ*B: nuclear factor kappa *B*; TNF-*α*: tumor necrosis factor *α*; AMPK: adenosine monophosphate-activated protein kinase; AST: aspartate aminotransferase; GLUT4: glucose transporter type 4; AchE: acetylcholinesterase; *MAO*: monoamine oxidase; ROS: reactive oxygen species; PKC: protein kinase C; JNK: Jun N-terminal kinase; ALT: alanine transaminase; TTG: tissue transglutaminase antibody.

*Artemisia* spp. have a broad range of pharmacological activities such as antiulcer, anticancer, hepatoprotective, antidiabetic, antioxidant, and antimicrobial. Some of the preclinical studies of these species' activities are summarized in Table 3 and Figure 2.

One of the available famous drugs derived from *Artemisia* species is artemisinin which exists in the leaves and flowers of *A. annua*. Other species which are known free of artemisinin were found to be active against malaria as *A. vulgaris*, *A. absinthium*, *A. dracunculus*, and *A. scoparia*; this activity was attributed to EOs and other sesquiterpenes [89]. Moreover, other studies also mentioned that artemisinin was not the only antimalarial substance in *A. annua* extracts [90–92].

### 5.1. Antioxidant

Antioxidants are a group of compounds that can help support the integrity of cells in the face of free radicals, unstable molecules that our body inevitably produces [93–95]. Natural antioxidants are thus essential for the proper functioning of the body [96–98]. Several studies have been reported the antioxidant activity of *A. absinthium*. Phenolic compounds (gallic acid, coumaric acid, vanillic acid, syringic acid, and chlorogenic salicylic acid) and flavonoids (quercetin and rutin) present in *A. absinthium* showed the potential of this plant against diseases related to oxidative stress [99–101]. These compounds reduce lipid peroxidation (thiobarbituric acid-reactive substances (TBARS) and recover endogenous antioxidant (e.g., superoxide dismutase (SOD) and glutathione (GSH)).

### 5.2. Anti-inflammatory

Inflammation is the body's natural response to protecting itself and recovering from an injury [102, 103]. It has the function of protecting the body from harmful substances and regenerating the damaged tissue [1, 104, 105]. *A. absinthium* extracts exhibit anti-inflammatory properties which may be linked to its secondary metabolites including flavonoids and sesquiterpene-type compounds and their role in inflammatory regulator inhibition such as bradykinins, histamine, prostaglandins, and serotonin [106] and through suppression of proinflammatory mediator expression such as inducible nitric oxide synthase (iNOS), prostaglandin E-2 (PGE2), cyclooxygenase-2 (COX-2), factor nuclear factor-kappa-B (NF-*κ*B), and tumor necrosis factor-*α* (TNF-*α*) [11].

### 5.3. Anticancer

Cancer is a disease in which the body's cells grow uncontrollably, forming a tumor that can spread to different parts of the body [107–111]. The mechanism of the anticancer effect *A. absinthium* extract was due to the activation of the mitogen-activated protein kinase/extracellular signal-regulated kinase MEK/ERK signaling pathway, which in turn stimulates the mitochondrial pathway of caspase activation and regulates Bad and Bcl-2 family proteins, resulting in the apoptotic death of MCF-7 and MDA-MB231 human cancer cells [108].

### 5.4. Neuroprotective

Neurocerebral disorders, especially neurodegenerative ones, refer to several progressive brain syndromes that affect memory, thinking ability, behavior, and emotions [112–115]. *A. absinthium* has been shown to have neuroprotective effects on cerebral damage caused by reperfusion through its nicotinic and muscarinic action. The protective mechanism of ethanolic extract of *A. absinthium* may be due to its anticholinesterase activity as well as the ability to change the behavior of rats by restoring acetylcholinesterase (AChE) and monoamine oxidase (MAO) enzymes to near-normal activity [11]. The sesquiterpenoid dimer—caruifolin D—found in *A. absinthium* may be used for the treatment of neurodegenerative diseases such as Alzheimer's or Parkinson's due to its inhibitory action on the production of neuroinflammatory mediators in BV2 microglial cells and the reactive oxygen species (ROS) production; leading to inhibitory effects on the activations of protein kinase C (PKC) and c-Jun N-terminal kinase (JNK) [116].

### 5.5. Hepatoprotective

*A. absinthium* hydroalcoholic extract improves hepatic function and lowers oxidative stress indicators and consistently stimulates and preserves the structural morphology of the hepatocellular membrane, resulting in lower serum aspartate (ASAT) and alanine aminotransferase (ALAT) activity. The proposed hepatoprotective mechanisms include liver microsomal drug-metabolizing enzyme suppression, free radical scavenging activity, and calcium channel blockage [117].

### 5.6. Antidiabetic

Diabetes is a metabolic disease that causes excess blood glucose (hyperglycemia) [118]. This disease is incurable and once diagnosed requires lifelong treatment [119]. *A. absinthium* extracts showed an insulin-sensitizing action due to their role in adenosine monophosphate-activated protein kinase (AMPK) stimulation and glucose transporter type 4 (GLUT4) translocation to the cell surface of the muscle [120]. In diabetic rats treated with *A. absinthium*, the metabolic pathway shifted towards carbohydrate as a source of energy, preserving proteins and lipids while increasing their production, leading to preventing body weight loss [121].

### 5.7. Antimalarial

A sesquiterpene lactone, artemisinin, which is the main active ingredient in *A. annua* is used for the treatment of *Plasmodium* parasites; these parasites are characterized by their substantial hemoglobin uptake and digestion. This produces large quantities of free redox-active heme and free ferrous iron (Fe^2+^), which are assumed to be responsible for artemisinin's parasite specificity. Infected erythrocytes convert excess heme to hematin, which is toxic to the parasite due to oxidative damage and direct cell membrane rupture; but malarial parasites have evolved a detoxification mechanism that uses a biocrystallization process to convert hematin to the less toxic and inert crystallized hemozoin. Activated artemisinin has been shown to inhibit the development of hemozoin by alkylating heme. As a result, artemisinin's activator and target are both free heme from hemoglobin breakdown [122].

## 6. Clinical Studies

Long traditional usage and functional preclinical studies of different *Artemisia* species for the treatment of several diseases encouraged their clinical evaluation to support the evidence of their potential as antimalarial, antioxidant, anticancer, antinociceptive, anti-inflammatory, and antiviral agents [4].

### 6.1. Anti-inflammatory Activity

In a randomized double-blind clinical trial, oral treatments of 42 patients by Arthrem (supercritical CO_2_-extracted *A. annua*) at doses 150 mg and 300 mg or placebo twice daily for 12 weeks were tested for their efficacy on stiffness, pain, and functional limitations in osteoarthritis of the hip and knees. Results showed a significant decrease in visual analogue scale (VAS) score and improvement in WOMAC (Western Ontario and McMaster Universities Osteoarthritis) total score only at the low dose of 150 mg [154]. Afterwards, an open-label 6 month extension trial was proceeded to examine the safety of Arthrem in the long run (6 months). Results showed that Arthrem could be a safe and effective agent for osteoarthritis management [155]. In a similar study, 90 patients diagnosed with osteoarthritis applied 3% *A. absinthium* ointment, 3% *A. absinthium* liniment, or piroxicam gel (PG) on their knees for 4 weeks. Results showed that *A. absinthium* ointment revealed significant improvement in all parameters except for WTSS (total stiffness score), where *A. absinthium* liniment only reduced VAS and WTPS (total pain score) in week 4 with recurrence in week 6 when compared to PG that improved all the parameters with no recurrence [156]. Furthermore, in a randomized controlled clinical trial, 10 patients with Crohn's disease administrated dried *A. absinthium* powder (750 mg three times daily) along with their basic therapy for 6 weeks, where another ten patients served as the control group. Results showed a significant reduction in serum TNF-*α* level and Crohn's disease activity index (CDAI) scores as well as remission of symptoms in eight patients [157].

In a further clinical study, a nasal spray containing an extract of *A. abrotanum* mainly composed of EO (4 mg/mL) and flavonols (2.5 *μ*g/mL) was established for treating 12 patients with allergic rhinitis. The EO fraction is composed of 1,8-cineole, linalool, and davanone, while the flavonol fraction contained centauredin, casticin, and quercetin dimethyl-ethers, which are well known for their anti-inflammatory effect. Most of the patients exhibited a significant reduction in nasal congestion, sneezing, and rhinorrhea as well as relief of eye symptoms when compared to the effect of anti-histamines [158].

In the sight of cosmetics, twenty-five sensitive skin patients were selected for investigating the efficacy of *A. annua* extract on skin reliving. Results showed that consuming cosmetics having *A. annua* extract for 4 weeks could improve the hydration degree of the cheek cuticle by 63.90%, reduce the transepidermal water loss by 21.51%, reduce the sensitized area by 77.47%, repair skin damage, and reduce redness [159].

### 6.2. Anticancer Activity

Interestingly, artemisinin and its derivatives were reported as potent antitumor compounds with high selectivity on cancer cells without any side effects on normal cells [160]. Their mechanism of action is based on the cleavage of their endoperoxide bridge by Fe^2+^ in cancerous cells and the production of ROS involved in apoptosis, DNA damage, autophagy, and cell cycle arrest G0/G1 of the cancerous cells. Additionally, they can suppress angiogenesis by inhibiting the secretion of vascular endothelial growth factor (VEGF), vascular endothelial growth factor receptor 2 (VEGFR2), and kinase insert domain receptor (KDR)/flk-1 in tumors, as well as affecting different signaling pathways and transcription factors related to tumor growth [161, 162]. Besides the antitumor effect of artemisinin and its derivatives against human cancer cell lines *in vitro* [160, 163, 164], different clinical studies were established to ensure their potency. For instance, in a pilot clinical trial at phase II, the anti-tumor effect of dihydroartemisinin (200 mg/day) was tested against advanced cervical carcinoma for 3 weeks in ten women. Results showed a significant reduction in vaginal discharge and pain, with no sign of severe toxicity, as well as improvement in overall signs and symptoms. These patients also exhibited a lower expression of epidermal growth factor receptor (EGFR) and Ki-67 oncogenes [165].

Further, in a randomized, controlled clinical trial, artesunate (120 mg, once daily, IV) was combined with vinorelbine (25 mg/m^2^, once daily, IV) and cisplatin (25 mg/m^2^, once daily, IV) to treat patients with the advanced lung cancer stage. Results showed significant improvement in the survival rate and hindering of the progression time of the cancerous cell, devoid of extra side effects [166]. In a case report conducted by Singh and Verma [167], artesunate (50 mg) proved to reduce the tumor size of the larynx of a patient with stage II cancer by 70% following 2 weeks of treatment [167]. As well in another case report conducted by Berger et al. [168], the combination of artesunate (50 mg twice/day) with standard chemotherapy showed a significant reduction in death risk and stabilization of the disease case in 2 patients with metastatic uveal melanoma stage IV, when compared to the chemotherapy alone [168]. Furthermore, artemether (40 mg once/day) proved to exhibit a significant improvement in the computed tomography scan of a 75-year-old patient with pituitary macroadenoma. Results showed a decrease in the tumor's density as well as an improvement in the overall clinical signs and symptoms [169].

### 6.3. Antidiabetic Activity

In a randomized double-blind clinical study, 24 patients with impaired glucose tolerance (IGT) administrated *A. dracunculus* (1000 mg) or placebo before breakfast and dinner for 90 days. Results showed a significant decrease in systolic blood pressure, glycated hemoglobin, and total insulin secretion as well as a significant increase in the high-density lipoprotein cholesterol level [170].

In a dose-response clinical study, ethanolic extract of *Artemisia princeps* Pamp. containing eupatilin and jaseocidin was investigated for its antidiabetic effect in 81 patients with hyperglycemia. Patients were randomized into four groups: negative control (lactose 2000 mg/day), positive control (pinitol 1140 mg/day), low-dose extract (2000 mg/day), and high-dose extract (4000 mg/day). Both doses significantly reduced glycated hemoglobin level, where the free fatty acid level in plasma was only lowered in patients administrated the high dose of the extract [171]. In a similar clinical trial, *A. absinthium* capsules (1 g/twice daily for 30 days) or placebo were administrated to 16 patients with type II diabetes. Results showed that *A. absinthium* reduced the glucose level by 32%, triglycerides by 10%, total cholesterol by 5%, and LDL level by 6% [172].

### 6.4. Antimalarial Activity

Malaria is considered the most common tropical disease that is provoked by certain parasites of the genus *Plasmodium* such as *P. malariae*, *P. viva*, *P. falciparum*, and *P. ovale* [173]. *Artemisia* species are famous for their content of sesquiterpene lactones that are responsible for the high therapeutic potential of the genus [173, 174]. For instance, artemisinin and its derivatives are the most common sesquiterpene lactones among the genus. Dihydroartemisinin (the active form inside the biological systems) is produced by reducing the lactone of artemisinin. While, alkylation of the hemiacetal group yields arteether and artemether, where artesunate is produced by acylation of the hemiacetal group with succinic acid [175]. Inside *in vivo* systems, all these derivatives are converted to dihydroartemisinin, where it possesses the highest activity, oral bioavailability, and tolerability with minimal side effects. These compounds are well known for their powerful activity against different species of *Plasmodium* as they contain the 1,2,4-trioxane moiety that may be responsible for the mechanism of action of the drugs [122]. The antimalarial activity of these compounds outcomes from the presence of Fe^2+^ after the *Plasmodium* hemolysis. This Fe^2+^ is utilized as a catalyst to open the peroxide bridge of the compound, leading to the formation of free radicals, alkylating *Plasmodium* proteins, and finally causing parasite death. Artemisinin also can perform its antimalarial activity by inhibiting PfATP6, an enzyme for the delivery of Ca^2+^ into vesicles of the parasite, which is critical for its development [122]. In a cluster-randomized clinical trial performed in different African countries, it was determined that rectal artesunate could take from 4 to 6 h to reduce parasite load, progression of the disease, and risk of death, so it can be a good choice for patients who cannot be treated orally [176]. Moreover, the WHO reviewed different clinical trials performed by the African Quinine Artesunate Malaria Trial multicentre on 5400 children under the age of 15 years with multidrug-resistant severe malaria and suggested IV artesunate (2.4 mg/kg once daily) as a choice to treat malaria [177].

A meta-analysis study using single-patient data from different randomized, controlled trials was conducted to compare artemether and quinine in treating severe *P. falciparum* infection. Results showed that the death rate was significantly reduced with patients treated with artemether (14%) when compared to patients treated with quinine (17%). On the other hand, there was no difference between the 2 treatments in coma recovery, fever clearance, or the progress of neurological toxicity. However, the overall adverse outcomes of either death or neurological toxicity were significantly fewer in the artemether-treated group [178].

In a double-blind, randomized, placebo-controlled clinical trial, artesunate was combined with sulfadoxine-pyrimethamine to test their efficacy in reducing the timing of malaria exposure during the infancy stage. Results showed that innate cells produced a balanced level of proinflammatory and regulatory cytokines around 2 years of age, which was accompanied by a lower risk of clinical malaria [179]. Further, it was reported that patients with uncomplicated malaria who administrated this combination therapy exhibited an 84.1% cure rate [180].

Despite the potency of artesunate as an antimalarial drug, there are also two main antimalarial regimen options: dihydroartemisinin–piperaquine (DHA–PPQ) and artemether-lumefantrine (AL), which are considered as the first option for treating uncomplicated *P. falciparum* malaria globally [181]. For instance, DHA–PPQ was reported to have high benefits in children with uncomplicated malaria in endemic countries [182]. On the other hand, the AL therapy (marketed as “Coartem”) could exert its antimalarial effect through opposing the erythrocytic stages of the parasite and so reducing the number of parasites. In addition, lumefantrine had a much-extended half-life time when combined with artemether and was supposed to clear residuals of the parasites [183].

As malaria causes severe maternal and fetal problems, the Centers for Disease Control and Prevention (CDC) suggested AL therapy for treating pregnant women with uncomplicated malaria in the United States throughout the second and third trimesters of pregnancy, at the same doses assigned for nonpregnant women [184]. In a case report implemented by Daddy et al. [185], dried leaves of *A. annua* were investigated for their efficacy as an antimalarial agent in patients (with *P. falciparum* infection) not responding to artemisinin combination therapy (ACT) or artesunate (IV). After oral administration of dried leaves of *A. annua* at a dose (0.5 g/twice daily/5 days), these patients exhibited a subside in clinical symptoms and the parasites were undetected microscopically [185]. In a large-scale double-blind, randomized clinical trial, the antimalarial effect of *A. annua* and *A. afra* infusions (1 L/day of dry leaf/twig infusion for 7 days) was compared to artesunate-amodiaquine (ASAQ) by 957 patients with malaria (with *P. falciparum* infection). Results showed that patients treated with both *Artemisia* infusions exhibited trophozoites clearance after 24 h when compared to ASAQ which took up to 14 days. Moreover, fever clearance took up to 48 h for ASAQ, but only 24 h for both *Artemisia* infusions. From days 14–28, gametocytes were undetectable for patients treated with *Artemisia* infusions, whereas on day 28, ASAQ-treated patients stayed carriers for gametocytes. These results proved that *Artemisia* infusions could break the life cycle of malaria by eliminating gametocytes with better outcomes than ASAQ [186]. In a questionnaire performed in Kenya and Uganda (2011) to study the antimalarial effect of *A. annua* teas, results showed prosperous outcomes after treating about 3000 cases including 250 children and 54 women in the first trimester of pregnancy with malaria [187].

### 6.5. Antiviral Activity as an Approach for Treating COVID-19 Infection

Recently, transposing of medications already in clinical use is the therapeutic strategy for controlling SARS-CoV-2 (COVID-19) infection [188, 189]. The WHO has recommended *A. annua* as a promising remedy for the treatment of COVID-19; however efficacy and side effects are not determined yet [190] (www.ClinicalTrials.gov, Identifier: NCT04530617). Moreover, *A. annua* is one of the ingredients of Jinhua Qinggan granule (one of the remedies suggested in the therapeutic regimen of COVID-19 in China) [191]. Currently, a phase II clinical study is under its way to evaluate the efficacy of *A. annua* in inhibiting the replication of the SARS-CoV-2 virus in patients with high-risk factors such as diabetes and hypertension (www.ClinicalTrials.gov Identifier: NCT04530617). As well, researchers from Saudi Arabia have established a placebo-controlled trial for evaluating the effect of artesunate in patients with mild symptoms of COVID-19 (www.ClinicalTrials.gov Identifier: NCT04387240).

Scientific evidence of this strategy might be due to the promising anti-inflammatory, immunomodulatory, and antiviral properties of the bioactive compounds in different *Artemisia* species, either among the preclinical or the clinical levels [4]. For instance, 85 patients with SARS were selected for a clinical study and 62 patients received the consigned treatment combined with the traditional Chinese medicine (TCM) (one of its components is herba *Artemisia*), while 23 patients were assigned in the control group. Results showed that patients who received the combined TCM regimen daily for 3 weeks showed a significant decrease in the total score of symptoms, as well as improvement of the lung X-rays, hepatic function, quality of life, and total score of mental sentiment factors [192].

Recently, it was reported that 1250 medical staff in Tongxu County Hospital take one or more decoctions of TCM daily as well as burn *Artemisia argyi* H.Lév. & Vaniot in the hospital corridor to cut off the route of transmitting infection, where *A. argyi* was stated as one of the herbs that can be used for contagion prevention by aromatherapy [193]. Artemisinin and its derivatives proved to have promising activities as antiviral agents. For instance, artesunate (100 mg/day) was examined for its efficacy to treat a 12-year patient with human cytomegalovirus (HCMV) infection who exhibited resistance against assigned antiviral drugs (foscarnet and ganciclovir) after stem cell transplantation. Results showed a significant reduction in viral load at day 7, with a virus half-life of 0.9–1.9 days, representing an effective stopover in viral replication [194].

## 7. Safety Issues of Artemisi*a* Species

Various reports have been published regarding toxicity related to the overdosing in humans with extracts of *Artemisia*; *A. absinthium* (wormwood) was used in the formulation of the absinthe drink and currently has been restricted in most countries because of neurotoxicity [195, 196]; it was reported that few cups of sage tea or wormwood would be essential to reach the suitable daily intakes [195]. Preclinical studies of the toxicity of *Artemisia* species were examined in many reports and the most important results were summarized in Table 4. Regarding *Artemisia* spp. toxicity, different parts of *Artemisia spicigera* K. Koch and *Artemisia fragrans* Willd. significantly increased the number of MCF7 and HEK293 cell proliferation [126]. Moreover, the toxicological study of *Artemisia judaica* L. has been studied by Nofal et al. [197] and observed acute and chronic toxicity. Furthermore, *Artemisia parviflora* Roxb. ex D. Don showed no significant toxic effect on Swiss albino mice [198] as shown in Table 4. In addition, there is only one report on the toxicity of *A. vulgaris* [199] while different studies of *A. annua* demonstrated that it is considered safe and nontoxic up to 5000 mg/kg of the extract [200]. Artemether and the closely related compound arteether are hydrophobic derivatives of dihydroartemisinin reported to have neurotoxicity action [201].

## 8. Limitations, Therapeutic Perspectives, and Clinical Gaps

Although *Artemisia* spp. and its constituents show great potential as functional foods, dietary supplements, and safe medicines, some adverse effects have been described in the literature [206]. *A. absinthium*, grand wormwood, has been included traditionally as a major component of the highly anise-flavoured alcoholic spirit, “Absinthe,” which was the most popular alcoholic beverage of the late 19^th^ century in Europe [207]. Absinthe was prohibited at the beginning of the 20^th^ century as a consequence of adverse symptoms called absinthism [208]. Absinthism symptoms included hallucinations, blindness, mental deterioration, and convulsions. The prebanned Absinthe was probably related to chronic alcoholism [207]. Several drugs can interact with the effects produced by Artemisinin, and therefore, the specialist should be consulted before taking it. *A. absinthium* is permitted nowadays in foods and alcoholic beverages. The consumption of thujone from *Artemisia* must not exceed 10 mg/kg according to the European Food Safety Authority (EFSA) and 3 mg/day/person according to European Medicines Agency (EMA) [206].

Some *Artemisia* species are used in regulating fertility and thus should be avoided in pregnancy due to the possible risk of embryotoxicity at higher doses. For instance, the consumption of *A. herba-alba* to pregnant mice significantly decreased the fertility ratio of offspring mice [209]. Additionally, consumption of *A. kopetdaghensis* “Krasch., Popov & Lincz. ex Poljakov” hydroalcoholic extract in pregnancy increases the risk of abortion [210].

Skin contact dermatitis has been reported upon exposure to different *Artemisia* species [211, 212]. Skin prick testing showed that the majority of patients with allergic rhinitis and asthma have positive reactions to *A. vulgaris*. Therefore, patients with *Compositae* sensitization are routinely warned against exposure to *Artemisia* species [213]. Artemisia-induced dermatitis is attributed to the content of sesquiterpene lactones [214].

Pollens from *Artemisia* species can cause serious pollinosis [206]. Nasal challenge and bronchial provocation tests verified that pollens, leaves, and stems from *Artemisia* are serious allergens causing allergic rhinitis and/or asthma [215, 216]. For instance, pollens from mugwort, *A. vulgaris*, contained allergenic substances such as profiling as well as other crossreactive allergens with immunoglobulin E (IgE) reactivity causing immediate type I allergic reactions [217]. Type I hypersensitivity involves mast cell degranulation and the release of inflammatory mediators such as histamine, causing allergic reactions such as anaphylactic shock [218]. Additionally, *A. vulgaris* pollens showed the highest levels of endotoxin among other collected plants across 100 locations in Europe [219]. Pollen extracts of six different *Artemisia* species, *A. annua*, *A. scoparia*, *A. vulgaris*, *A. princeps*, *Artemisia campestris* L., and *Artemisia tridentata* Nutt. exhibited an extensive degree of similarity and crossreactivity [220]. This study also showed that Korean and Norwegian patient sera had the same pattern of reactivity towards *A. vulgaris* and *A. princeps* [220].

Clinically, administration of the sesquiterpene lactone artemisinin as well as its derivatives such as arteether, artesunate, and artemether in appropriate therapeutic doses for short periods did not show serious side effects [221, 222]. In the liver, artemisinin is converted to various inactive metabolites, such as deoxy artemisinin, deoxyhydroartemisinin, crystal 7, and 9,10-dihydrodeoxyarthemisinin. The reaction is catalyzed by a CYP2B6 enzyme, while another CYP3A4 enzyme acts as a secondary catalyst. These enzymes belong to the cytochrome P450 group present in the smooth endoplasmic reticulum. Artemisinin derivatives are metabolized differently. They are first converted to dihydroartemisinin (DHA). DHA itself is a powerful antimalarial molecule and is active in the bloodstream for two to three hours [223]. The antimalarial activity of artesunate is actually only through DHA [224]. Artesunate is converted to DHA within one minute of absorption. About 90% of all DHA is normally bound to blood plasma. In the liver, the cytochrome P450 enzyme system (including CYP2A6, CYP3A4, and CYP3A5) converts DHA into inactive metabolites [225]. All metabolites are subject to glucuronidation and are excreted in the urine or faeces. UDP-glucuronosyltransferases, especially UGT1A9 and UGT2B7, are responsible for the process [226]. DHA is also excreted in the bile as minor glucuronides, such as tetrahydrofuran acetate. Due to its rapid metabolism, artemisinin is a relatively safe bioactive compound [227]. Because artemisinin interactions with other drugs are not fully known, future clinical studies are needed to establish their potential interaction mechanisms.

Other limitations derive from the amounts of active ingredients in plants, depending on the area of cultivation and climate [228–230]. Also, the absorption of bioactive compounds rapidly and in variable quantities requires the future development of pharmaceutical nanoformulations that will improve the bioavailability and implicitly an increased therapeutic efficacy [231, 232].

## 9. Overall Conclusion

*Artemisia* spp. have been traditionally used for pharmacological purposes and as an edible plant used in food, spices, and beverages. *A. annua* and *A. absinthium* are the most famous *Artemisia* species. This genus distributed worldwide presents diverse chemical constituents mainly EOs and polyphenols. These species contain sesquiterpene lactones that are largely responsible for the therapeutical potential of *Artemisia* genus. The most studied biological activities of this genus are antioxidant, anti-inflammatory, antitumor, antidiabetic, antimalarial, neuroprotective, and hepatoprotective activities through preclinical and clinical evidence. *Artemisia* spp. and their constituents show great potential as dietary supplements, functional foods, and safe medicines as antimalarial, antioxidant, anticancer, antinociceptive, anti-inflammatory, and antiviral agents. The antiviral activity for treating COVID-19 infection is a hope for the current pandemic. However, it is really important and necessary for further research investigations for discovering safer *Artemisia* plant-derived drugs for curing several kinds of diseases.

## Figures and Tables

**Table 1:** Essential oil composition of the *Artemisia* genus from different geographical regions (2017–2021).

Plant species	Parts used	Chemical composition	Region/country	References
*Artemisia absinthium* L.	L	Camphor; E-caryophyllene; eucalyptol; germacrene D; *α*-cadinol	Brazil	[39]
*Artemisia anethoides* Mattf.	AP	1,8-Cineole; terpinen-4-ol; 2-isopropyltoluene; pinocarveol	China	[40]
*Artemisia annua* L.	AP	Artemisia ketone; *α*-caryophyllene; germacrene D	China	[41]
	AP, F, L	(E)-*β*-Farnesene; germacra-4(15),5,10(14)-trien-1-ol; *Artemisia* alcohol 3-methyl butanoate; yomogi alcohol; *Artemisia* alcohol 3-phenylpropionate; *Artemisia* alcohol 2-methyl butanoate; *α*-copaene; artemisia alcohol; 1,8-cineol	Russia	[42]
*Artemisia arborescens* (Vaill.) L.	F, L	*β*-Thujone, camphor, terpinen-4-ol, germacrene D, chamazulene	Italy	[43]
*Artemisia argyi* H. Lév. & Vaniot	L	*α*-Thujone; bornanone; terpinen-4-ol; cis-2-menthenol; borneol; cis-sabino; *α*-terpineol; *β*-caryophyllene; caryophyllene oxide; neointermedeol	China	[44]
*Artemisia campestris* L.	—	*β*-Pinene; cadin-4-en-7-ol; Z-*β*-ocimene; *γ*-terpinene	Portugal	[45]
	AP	*β*-Pinene; *α*-pinene; myrcene; germacrene D; (Z)-*β*-ocimene; *γ*-curcumene	Algeria	[46]
	AP	*β*-Pinene; spathulenol; *α*-pinene; limonene; o-cymene	Morocco	[47]
	L, S	*β*-Pinene; 2-undecanone; limonene; benzene; *α*-pinene; 1,4-cyclohexadiene; *β*-myrcene; 2-naphthalenemethnol; 2-decanone	Tunisia	[48]
*Artemisia dracunculus* L.	AP	p-Allylanisole; ocimene (e)-*β*; ocimene (z)-*β*; limonene	Iran	[49]
	L	Methyl eugenol; elemicin; isoelemicin; (*Z*)-methyl isoeugenol	Poland	[50]
*Artemisia gmelinii* Weber ex Stechm.	—	*γ*-Amorphene; isohumbertiol B; caryophyllene oxide; caryophylla-4 (12), 8(13)-diene-5*α*-ol; ylangenol; caryophyllene; cabrevia oxide B	Russia	[51]
	AP	Cyclobutane ethanol; endo-borneol; germacrene D; eucalyptol; selin-6-en-4*α*-ol; bisabolone oxide A; caryophyllene; terpinen-4-ol	China	[52]
	AP	Phellandrene; ascaridole; *α*-terpinolene; isoascaridole; benzyl isovalerate	India	[53]
*Artemisia herba-alba* Asso	AP	cis-Thujone; trans-thujone; vanillyl alcohol; nordavanone; cis, threo-davanafuran	Morocco	[54]
	AP	3-Thujanone a; 3-thujanone b; camphor	Sweden	[55]
	L	*α*-Thujone; germacrene D; 1,8-cineole; *β*-thujone	Tunisia	[56]
*Artemisia jordanica* Danin	L	2,3-Dehydro-1,8-Cineole; camphene; endo-borneol; bornyl acetate; geranyl isovalerate	Palestine	[57]
*Artemisia judaica* L.	AP	Methyl pentanoate; (E)-salvene; santolina triene; allyl isovalerate; *α*-pinene; *β*-citronellene; camphene; benzaldehyde; myrcene; mesitylene; yomogi alcohol; 1,4-cineol; *α*-terpinene; artemisia ketone; 2,6-dimethyl phenol; chrysanthenone; camphor; artemisyl acetate; piperitone; (Z)-ethyl cinnamate; (E)-ethyl cinnamate; germacrene D; davanone	Jordan	[58]
	AP	Butanoic acid; *β*-linalool; 2-cyclohexen-1-one, 3-methyl-6-(1-methylethyl); acenaphthene; davana ether	Saudi Arabia	[59]
*Artemisia magellanica* Sch.Bip.	AP	*γ*-Costol; (Z)-en-yn-dicycloether; *α*-selinene; selina-4,11-diene (eudesma-4,11-diene); (E)-*β*-farnesene; 2-methylbutyl 2-methylbutyrate; (Z)-*β*-ocimene	Argentina	[60]
*Artemisia monosperma* Delile	AP	Spathulenol; cloven; *β*-linalool; *α*-citral; geranyl acetate; isohomogenol; benzene, 1,2-dimethoxy-4-(1-propenyl); caryophyllene; aristolene; 2-propenoic acid, 3-phenyl-ethyl ester	Saudi Arabia	[59]
	L	*β*-Pinene; limonene; cis-*β*-ocimene; *α*-terpinolene; cis-sabinene hydrate; bornyl acetate	Saudi Arabia	[61]
*Artemisia nilagirica* (C.B.Clarke) Pamp.	AP	*β*-Thujone; germacrene-D; *β*-thujone; caryophyllene; caryophyllene oxide; borneol	India	[62]
*Artemisia pedemontana* Balb.	F, L	*α*-Pinene; camphene; p-cymene; 1,8-cineole; linalool; camphor; borneol; terpinen-4-ol; viridiflorol; 1-*α*-terpineol	Spain	[63]
*Artemisia persica* Boiss.	AP	Laciniata furanone E; artedouglasia oxide C; pinocarvone; trans-pinocarveol; *α*-pinene; 1,8-cineole; artedouglasia oxide B and D	Iran	[64]
*Artemisia sieversiana* Ehrh.	AP	Santolina triene; *α*-thujone; eucalyptol; *α*-sabinene; trans-2-menthen-1-ol; *α*-selinene, caryophyllene epoxide	China	[65]
*Artemisia tournefortiana* Rchb.	—	cis-Spiroether; Z-*β*-farnesene; trans-nerolidol; camphor	India	[66]
*Artemisia scoparia* Waldst. & Kitam.	AP	Spathulenol; acenaphthene; davana ether; 2-propenoic acid, 3-phenyl-ethyl ester	Saudi Arabia	[59]
*Artemisia sieberi* Besser	AP	Acenaphthene; 2-cyclohexen-1-one, 3-methyl-6-(1-methylethyl)	Saudi Arabia	[59]
*Artemisia vulgaris* L.	AP	Caryophyllene; humulene; germacrene D; borneol; caryophyllene oxide	Brazil	[67]
	AP	Germacrene D; 1,8-cineole; *β*-pinene; sabinene; cis-thujone; *β*-caryophyllene; caryophyllene oxide; *α*-humulene; davanone	Lithuania	[68]

AP: areal part; L: leaves; F: flowers; —: not reported.

**Table 2:** Chemical composition of *Artemisia* genus from different geographical regions.

Plant species	Chemical composition	Region/country	References
*Artemisia abrotanum* L.	Caffeic acid; chlorogenic acid; isochlorogenic acid; protocatechuic acid; rosmarinic acid; quercitrin	Saudi Arabia	[69]
*Artemisia absinthium* L.	Artemisinin; *α*-thujone; *β*-thujone; bornyl acetate; 4-terpineol; camphene; chamazulene; cadinene; myrcene; guaiazulene; linalool; *γ*-terpinene	-	[11]
*Artemisia annua* L.	Arteannuin B; artemisinin; artemisinic acid; scopoletin	China	[70]
*Artemisia campestris* L.	Catechin; vanillic acid; caffeic acid; syringic acid; p-coumaric acid; gallic acid	Morocco	[71]
*Artemisia capillaris* Thunb.	Neochlorogenic acid; chlorogenic acid; cryptochlorogenic acid; caffeic acid; 1,3-dicaffeoylquinic acid; 3,4-dicaffeoylquinic acid; 3,5-dicaffeoylquinic acid; 4,5-dicaffeoylquinic acid	China	[72]
*Artemisia dubia* L. ex B.D.Jacks.	Calotropoleanyl ester; *α*-amyrin; nonacosanoic acid; docosanoic acid; tetracosanoic acid; 1-(O-tricosanoyl) glycerol; 1-(O-pentacosanoyl) glycerol; *β*-sitosterol	Pakistan	[73]
*Artemisia gmelinii* Weber ex Stechm.	Coumarins; phenolics; flavonoids; caffeoylquinic acids; diterpene glycosides	Korea	[74]
*Artemisia herba-alba* Asso	Camphor; hanphillin; alkhanin; terpinen-4-ol; *α*-santonin; *α*-thujone; *β*-thujone; 2,5-bornanedione	Morocco	[75]
*Artemisia indica* Willd.	5-Hydroxy-3,7,4′-trimethoxyflavone; ludartin; maackiain; lupeol; cis-matricaria ester; trans-matricaria ester; 6-methoxy-7,8-methylenedioxy coumarin	China	[76]
*Artemisia lactiflora* Wall. ex DC.	*β*-Sitosterol; daucosterol; umbelliferone; isofraxidin; scopoletin; fraxidin; mandshurin; fraxin-8-O-*β*-D-glucopyranoside; euoniside; scopolin; 5,8-dihydroxy-7,4′-dimethoxy-flavone; syringing; chrysoeriol; tricin; luteolin; acacetin; apigenin; 5,7-dihydroxy-3,6,4′-trimethoxy-flavone; tectorigenin; eicosyl/docosyl-*p*-coumarate; isoferulic acid; ferulaldehyde; ethyl caffeate; caffeic acid; (−)-syringaresinol; (+)-diasyringaresinol; *p*-hydroxybenzoic acid; *p*-methylbenzaldehyde; cleomiscosin C; cleomiscosin A or B; biisofraxidin	China	[77]
*Artemisia mongolica* (Fisch. ex Besser) Fisch. ex Nakai	1-(3-Hydroxyphenyl)-2-(5-hydroxy-3-methoxyphenyl)ethane; 1-(3-hydroxyphenyl)-2-(3,5-dihydroxyphenyl)ethane	China	[78]
*Artemisia myriantha* Wall. ex Besser	Blumenol A; (+)-dehydrovomifoliol; (+)-3-hydroxy-*β*-ionone; (3R, 6R, 7E)-3-hydroxy-4, 7-megastigmadien-9-one; (−)-10-oxo-isodauc-3-en-15-oic acid; isoerivanin; eudesmafraglaucolide; artanomalide A; 13-acetoxy-3*β*-hydroxy-germacra-1(10) E,4E,7(11)-trien-12,6*α*-olide; 13-acetoxy-3*β*-tigloyl-germacra-1(10) E, 4E, 7(11)-trien-12, 6*α*-olide (10),13-acetoxy-3*β*-(3-methylbutanoyl)-germacra-1(10)E, 4E, 7(11)-trien-12, 6*α*-olide (11),3,9-diacetoxy-13-hydroxy-1(10), 4, 7(11)-germacratrien-12,6*α*-olide; 8*α*-angeloyloxycostunolide	China	[79]
*Artemisia pontica* L.	n-Hexadecanoic acid; 9,12,15-(Z,Z,Z)-octadecatrienoic acid; 2-(4a,8-dimethyl-7-oxo-1,2,3,4,4a,7-hexahydronaphthalen-2-yl)-propionic acid; 8-nitro-(1H)quinolin-4-ol-2-one; neophytadiene	Ukraine	[80]
*Artemisia rupestris* L.	Citrusin A; alaschanioside A; coniferin; citrusin B; syringaresinol-*β*-D-glucoside; (6R,9S)-3-carbonyl-*α*-ionol glucopyranoside; byzantionoside B	China	[81]
*Artemisia scoparia* Waldst. & Kitam.	Eugenol; capillene; spathulenol; capillin; scoparone; tricosane; heptacosane; nonacosane; stigmasterol; tritriacontane	Serbia	[82]
	4-Pyridone glucoside; polyacetylene glucosides	China	[83]
	Quercetin-3-O-*β*-d-glucoside; 3,4-dihydroxy-5-methoxycinnamic acid; caffeic acid; 6,7 dimethoxycoumarin	China	[84]
*Artemisia splendens* Willd.	Narcisin; quercetin; luteolin; kaempferol; genkwanin; astragalin; isorhamnetin-3-O-*β*-D-glucoside	Iran	[85]
*Artemisia turanica* Krasch.	3,5-Dicaffeoylquinic acid; 4,5-dicaffeoylquinic acid; 3,5,3′,4′-tetrahydroxy; 7,5′-methoxy flavones	Iran	[86, 87]
*Artemisia vulgaris* L.	Artanoic acid; luteolin; 6-methoxyluteolin; eupatilin; o-coumaric acid; vanillic acid; protocatechuic acid; 4-hydroxyphenyl acetate; vulgarin	Vietnam	[88]

**Table 3:** Preclinical pharmacological studies of different *Artemisia* species.

Extract/compound	Doses	*In vitro/in vivo*	Route of administration/assay	Model/cells	Activity	Potential effect	Reference
*A. nilagirica/*ethanolic extracts	500 mg/kg	*In vivo*	Orally	Rats	Antiulcer	Gastroprotective, ↑proteins of mucus content	[123, 124]
*A. nilagirica/*methanolic extract	150–250 mg/kg	*In vivo*	Orally	Swiss albino mice		Gastroprotective compared to standard drug vincristine	[125]
*A. absinthium, A. vulgaris/flowers/*methanolic extract	62.5, 125, 250, 500 *μ*g/mL	*In vitro*	MTT	MCF7 cells	Anticancer	↑cytotoxicity IC_50_ = 221–500 *μ*g/mL	[126]
*A. nilagirica/*ethyl acetate, hexane fractions	100 *μ*g/mL	*In vitro*	SRB	DLD-1 cells		↑cytotoxicity IC_50_ = 15.42–23.4 *μ*g/mL	[127]
*A. vulgaris/leaves/*methanolic extract	0.01–1.0 mg /mL	*In vitro*	MTT	Hepatocellular carcinoma cells		↑apoptosis IC_50_ = 0.1 mg/mL	[15]
*A. absinthium/*methanolic extract	20, 25 g/mL	*In vitro*	MTT	MCF-7 MDA-MB231		↑cancer cells suppression	[108, 128]
*A. armeniaca/*CH_2_Cl_2_ fraction	6.25–200 *μ*g/mL	*In vitro*	MTS	Apoptosis-proficient HL60 apoptosis-resistant K562		HL-60: IC_50_ = 75 *μ*g/mL, K562: IC_50_ = 130 *μ*g/mL	[129]
*A. dracunculus/*aerial parts, roots/ethanol, aqueous extracts	250 mg/kg	*In vivo*	Orally	STZ-induced diabetic rats	Antidiabetic	↓TGL, ↓LDL, ↓HDL	[14]
*A. dracunculus* L. (PMI 5011)/ethanolic extract	PMI 5011 (1%)	*In vivo*	Diet	KK-A^y^ mice		↑sensitivity of insulin, ↑insulin receptor signaling	[130, 131]
*A. sieberi* (*A. herba-alba*)/aqueous extracts	0.39 g/kg	*In vivo*	Orally	Alloxan-induced diabetic rats		↓blood glucose, ↑RBC, ↑WBC, ↑PCV, ↑ESR, ↑neutrophils, ↓heart rate	[132]
*A. persica/*aqueous, methanolic extracts	300, 400, 500 mg/kg	*In vivo*	Orally	Sprague-Dawley rats	Antihypertensive	↓systolic blood pressure in normotensive/hypertensive rats	[133]
*A. absinthium/*aqueous extract	50, 100, 200 mg/kg	*In vivo*	Orally	Kunming mice, NIH mice	Hepatoprotective	↓inflammatory cells, ↓liver lipid peroxidation, ↑SOD, ↑GPx	[134]
*A. vulgaris/*aerial parts/crude extract	150, 300, 600 mg/kg	*In vivo*	i.p.	Balb-C mice		↑liver structure, ↓parenchyma congestion, ↓cellular swelling, ↓apoptotic cells	[16]
*A. nilagirica*/leaf extracts	32–512 *μ*g/mL	*In vitro*	Agar disk diffusion method	15 bacterial strains	Antibacterial	Methanol, hexane extracts, ↑inhibition against phytopathogens	[135]
*A. herba- alba*, *A. judaica*, *A. monosperma/*EO	10.0, 5.0, 2.5, 1.0, 0.5 *μ*L/disc	*In vitro*	Agar disc diffusion method.	*Staphylococcus aureus* ATCC29213, *Escherichia coli* ATCC 25922		IC_50_ = 0.5–2.5 *μ*L*A. judaica*, A*. monosperma* plants had the highest MIC	[136]
*A. judaica/*ethanol extract	250, 500, 1000, 2000, 4000 *μ*g/mL	*In vitro*	(mic90) growth inhibition	Protozoan parasite (blastocystis)	Antiprotozoal	IC_5_ = 4000 *μ*g/mL, ↓growth, ↑destruction of blastocystis	[137]
*A. nilagirica/*EO	0.33 *μ*L/mL	*In vitro*	Inverted petri plate technique	*A. flavus*, *A. niger*, *A. ochraceus*	Antifungal	IC_50_ = 1.6 *μ*L/mL, ↓fungal growth, ↓mycotoxin secretion, ↓aflatoxigenic, ↓ochratoxigenic strains	[138]
*A. annua/l*eaves/EO ethanolic extract	EO = 470 mg/kg ethanol extract = 450 mg/kg	*In vivo*	i.p.	Wistar rats	Antidepressant	↑immobility time in the FST, ↓other activities in the OFT depressors of SNC	[139]
*A. absinthium/*aerial parts/methanolic extract	125, 250, 500, 1000 mg/kg	*In vivo*	i.p.	Swiss albino mice		↓immobility period in the fst and tst.dose-dependent antidepressant activity	[140, 141]
*A. vulgaris/*leaves/methanolic extract	50, 100, 300 mg/kg	*In vivo*	i.p.	Swiss albino mice	Antiepileptic	Anticonvulsant activities were noticed using EPM and MBT	[18]
*A. capillaris/*herba/ethanolic extract	50, 100, 200, 400 mg/kg	*In vivo*	Orally	Mice		Anticonvulsivant effect through the GABA-ergic neuron	[142]
*A. nilagirica*/leaves/ethanolic, aqueous extracts	100, 200 mg/kg	*In vivo*	i.p.	Swiss albino mice	Anti-Alzheimer	Confirmation of the anti-Alzheimer's activity of ethanol extract after object recognition and y-maze tests	[143]
*A. nilagirica/*leaves/ethanolic, aqueous extracts	100, 200 mg/kg	*In vivo*	i.p.	Swiss albino mice	Anti-Parkinson	↓catalepsy score in animals treated with ethanolic extract, ↑locomotor activity, ↑rotarod readings	[143]
*A. annua/*aqueous, ethanolic extracts	2 g/L	*In vitro*	ABTS, ORAC, FRAP	—	Antioxidant	↑protection against the oxidative deterioration of oil-in-water emulsion	[144]
*A. dracunculus* L./leaves/methanolic extract	20 *μ*L	*In vitro*	DPPH	—		↑antioxidant activity by phenolics	[13]
*A. nilagirica/*leaves/ethanolic, aqueous extracts	50–250 *μ*g/mL	*In vitro*	DPPH	—		Antioxidant activity of ethanolic extract > aqueous extract	[143]
*A. scoparia*, *A. spicigera/*methanolic extracts	0.25; 0.125; 0.0625; 0.0312; 0.0156; 0.0078; 0.0039; 0.0019; 0.0009; 0.00048 mg/mL	*In vitro*	DPPH	—		↑free radical scavenging activity RC_50_ = 0.03157, 0.0456 mg/mL	[145]
*A. nilagirica/*EO	10; 8.6; 6.5; 6.5; 3.3; 2.5; 2 *μ*g/mL	*In vitro*	Method recommended by WHO	*Aedes albopictus* mosquito	Insecticidal	LC_50_ = 5 *μ*g/mL	[146]
*A. nilagirica/*EO, chloroform, petroleum ether methanolic extracts	—	*In vitro*	Method recommended by WHO	*Aedes aegypti*, A*nopheles stephensi*, *Culex quinquefasciatus* mosquito larvae		The EO of *A. nilagirica* was the most effective larvicide against *A. stephensi* larvae	[147]
*A. aucheri/*methanolic extract	25, 50, 100 mg/mL	*In vitro*	Scolicidal tests	*Echinococcus granulosus*		↓effect on the protoscolices of hydatid cysts	[148]
*A. vulgaris/*ethanolic extract	1, 5, 10, 50, 100, 500, 1000 ppm	*In vitro*	Method recommended by WHO	*Aedes aegypti*		LC_50_ = 65.8 ppm in 1 h and 18.6 ppm in 24 h, *↓ A. aegypti* in various stages of its lifecycle	[19]
*A. scoparia*, *A. spicigera/*n-hexane, DCM, MeOH extracts	20, 40, 80 mg/mL	*In vitro*	Toxicity bioassay	*Tribolium castaneum* (red flour beetle)		Insecticidal properties, ↑activity of DCM extract	[145]
*A. scoparia*/butanol fraction	20 mg/site	*In vivo*	Topically	BALB/C mice	Antiatopic dermatitis	↓clinical symptoms in a DNFB mouse model that induced lesions, ↓inflammatory cytokines	[149]
*A. scoparia/*aerial parts/methanolic extract	150, 300 mg/kg	*In vivo*	—	Sprague-Dawley rats	Nephroprotective	↓DNA damages, dose = 300 mg/kg, ↓oxidative stress	[150]
*A. capillaris* Thunb/extract	10 mg/mouse/day	*In vivo*	Topically	Dermatophagoides farinae-sensitized NC/NGA mice	Anti-inflammatory, anti-atopic dermatitis	↓dermatitis scores, ↓bleeding, ↓hyperkeratosis, ↓hypertrophy in the dorsal skin and ear of the epidermis, ↓histamine	[151]
*A. pallens/*aerial parts/methanolic extract	200, 400 mg/kg	*In vivo*	Orally	Wistar rats	Anti-inflammatory, antioxidative	↓level of hepatic enzymes, ↑renal antioxidant enzymes	[152]
*A. vulgaris*/leaf/ethanolic extract	250, 500, 750, 1000 mg/kg	*In vivo*	Orally	ICR mice infected with *P. berghei*	Antimalarial	*↓P. berghei*, nontoxic	[17]
*A. scoparia*, *A. spicigera/*dichloromethane extracts	0–2 mg/mL 10% DMSO	*In vitro*	Heme biocrystallization and inhibition assay			IC_50_ = 0.778 mg/mL, IC_50_ = 0.998 mg/mL	[145]
*A. annua/*aqueous, hydro alcoholic extracts	—	*In vitro*	Parasite lactate dehydrogenase (pLDH)	*Plasmodium falciparum*		IC_50_ = 3.27 nM, IC_50_ = 4.95 nM	[153]
*A. annua/*aqueous hydro alcoholic extracts	Aqueous extract 1000 mg/kg/day, hydro alcoholic extract 500 mg/kg/day	*In vivo*	-	*Plasmodium berghei* NK173-infected m ice		↑activity on malaria of artemisinin, both extracts of *A. annua* are effective on malaria	[153]

↑: increase; ↓: decrease; MTT: 3-(4,5-dimethylthiazol-2-yl)-2,5-diphenyl-2H-tetrazolium bromide; DCM: dichloromethane; DNFB: 2,4-*dinitrofluorobenzene*; EPM: elevated plus maze; ESR: erythrocyte sedimentation rate; EO: essential oil; FST: forced swimming test; HDL: high-density lipoprotein; LDL: low-density lipoprotein; MBT: marble-burying test; MIC: minimum inhibitory concentration; OFT: open-field test; PCV: packed cell volume; RBC: red blood cell; SRB: sulforhodamine B; TC: total cholesterol; TGL: triglycerides; WBC: white blood cell; WHO: World Health Organization: i.p.: intraperitoneally; FRAP: ferric-reducing ability of plasma; ABTS: 2,2′-azino-bis(3-ethylbenzothiazoline-6-sulfonic acid; ORAC: oxygen radical absorbance capacity.

**Table 4:** Toxicological studies of *Artemisia* species.

Extract/compound	Doses	*In vitro/in vivo*	Route of administration	Model	Effect	Ref
*A. annua* /leaf/hexane extract	1000, 2000, 2500 mg/kg	*In vivo*	i.p.	Wistar albino rats	↓carbohydrate, protein, lipid metabolisms, unfavourable effect on nutritional benefits, ↓hematological parameters, ↓toxicity when used acutely in rats	[202]
*A. annua/*hydroethanolic extract	300, 2000, 5000 mg/kg	*In vivo*	Orally	Swiss albino mice	No toxic or lethal reactions of all the doses	[200]
*A. parviflora*/aerial parts/ethanolic extract	0.10, 0.50, 1.0 g/kg	*In vivo*	Orally	Swiss albino mice	No significant toxic effect LD_50_ > 1 g/kg BW	[198]
*A. abyssinica*, *A. inculta*/aerial parts/ethanolic extracts	500 mg/kg, 1 and 3 g/kg	*In vivo*	Orally	Swiss albino mice	*A. inculta*, dose = 3 g/kg: CNS stimulation, *A. abyssinica*, dose = 3 g/kg: ↓locomotor activity	[203]
*A. vulgaris/*oils	—	*In vivo*	—	Brine shrimp *Artemia* sp. (larvae)	LC_50_ = 10.4–23.3 *μ*g/mL germacrene D, camphor, 1,8-cineol, davanone: ↑toxicity	[199]
*A. afra*/aqueous extract	1.5–5.5 g/kg i.t., 2–24 g/kg o.p.	*In vivo*	i.p., orally	BALB/C mice, Wistar rats	Nontoxic when given acutely, low chronic toxicity, hepatoprotective effect in high doses	[204]
Artemether	0, 20, 40, 80 mg/kg i.m., 0, 50, 150, 600 mg/kg p.o.	*In vivo*	i.m., orally	Beagle dogs	High i.m. doses: neurological damage, dose = 20 mg/kg: minimal effects occurred	[201]
Artemether, artesunate	30–100 mg/kg/day	*In vivo*	i.m.	Swiss albino mice	Artemether neurotoxicity is significantly more neurotoxic than i.m. artesunate	[205]

## Data Availability

The data supporting this review are from previously reported studies and datasets, which have been cited. The processed data are available from the corresponding author upon request.

## References

[1] Sharifi-Rad J., Quispe C., Kumar M. (2022). *Hyssopus* essential oil: an update of its phytochemistry, biological activities, and safety profile. *Oxidative Medicine and Cellular Longevity*.

[2] Su X. Z., Miller L. H. (2015). The discovery of artemisinin and the Nobel Prize in Physiology or Medicine. *Science China Life Sciences*.

[3] Ahuja A., Yi Y.-S., Kim M.-Y., Cho J. Y. (2018). Ethnopharmacological properties of *Artemisia asiatica*: a comprehensive review. *Journal of Ethnopharmacology*.

[4] Bora K. S., Sharma A. (2011). The genus *Artemisia*: a comprehensive review. *Pharmaceutical Biology*.

[5] Nigam M., Atanassova M., Mishra A. P. (2019). Bioactive compounds and health benefits of *Artemisia* species. *Natural Product Communications*.

[6] International Union for Conservation of Nature Global Species Programme Red List Unit. https://www.iucnredlist.org/search/list?taxonomies=115307&searchType=species.

[7] Ahuja J., Suresh J., Paramakrishnan N., Mruthunjaya K., Naganandhini M. N. (2011). An ethnomedical, phytochemical and pharmacological profile of *Artemisiaparviflora* Roxb. *Journal of Essential Oil Bearing Plants*.

[8] da Silva J. A. T. (2004). Mining the essential oils of the Anthemideae. *African Journal of Biotechnology*.

[9] Ryu B. K., Ahn B. O., Oh T. Y., Kim S. H., Kim W. B., Lee E. B. (1998). Studies on protective effect of DA-9601, *Artemisia asiatica* extract, on acetaminophen-and CCI 4-induced liver damage in rats. *Archives of Pharmacal Research*.

[10] Feng X., Cao S., Qiu F., Zhang B. (2020). Traditional application and modern pharmacological research of *Artemisia annua* L. *Pharmacology & Therapeutics*.

[11] Batiha G. E.-S., Olatunde A., El-Mleeh A. (2020). Bioactive compounds, pharmacological actions, and pharmacokinetics of wormwood (*Artemisia absinthium*). *Antibiotics*.

[12] Liu N., Van der Kooy F., Verpoorte R. (2009). *Artemisia afra*: a potential flagship for African medicinal plants?. *South African Journal of Botany*.

[13] Khezrilu B. J., Heidari R. (2014). The evaluation of antioxidant activities and phenolic compounds in leaves and inflorescence of *Artemisia dracunculus* L. by HPLC. *Journal Of Medicinal Plants*.

[14] Samyal M., Kumar H., Khokra S., Parashar B., Sahu R., Ahmed Z. (2011). Evaluation of antidiabetic and antihyperlipidemic effects of *Artemisia dracunculus* extracts in streptozotocin-induced-diabetic rats. *Pharmacologyonline*.

[15] Sharmila K., Padma P. (2013). Anticancer activity of *Artemisia vulgaris* on hepatocellular carcinoma (HepG2) cells. *International Journal of Pharmacy and Pharmaceutical Sciences*.

[16] Gilani A. H., Yaeesh S., Jamal Q., Ghayur M. N. (2005). Hepatoprotective activity of aqueous–methanol extract of *Artemisia vulgaris*. *Phytotherapy Research: An International Journal Devoted to Pharmacological and Toxicological Evaluation of Natural Product Derivatives*.

[17] Bamunuarachchi G. S., Ratnasooriya W. D., Premakumara S., Udagama P. V. (2013). Antimalarial properties of *Artemisia vulgaris* L. ethanolic leaf extract in a Plasmodium berghei murine malaria model. *Journal Vector-Borne Disease*.

[18] de Almeida E. R., da Silva A. R., Aragatilde A. C. (2013). Anticonvulsant and anxiolytic assessment of leaves from *Artemisia vulgaris* L. in mice. *Journal of Medicinal Plants Research*.

[19] Ninditya V. I., Purwati E., Utami A. T. (2020). *Artemisia vulgaris* efficacies against various stages of Aedes aegypti. *Veterinary World*.

[20] Bisht D., Kumar D., Kumar D., Dua K., Chellappan D. K. (2021). Phytochemistry and pharmacological activity of the genus *Artemisia*. *Archives of Pharmacal Research*.

[21] Ding J., Wang L., He C., Zhao J., Si L., Huang H. (2021). Artemisia scoparia: traditional uses, active constituents and pharmacological effects. *Journal of Ethnopharmacology*.

[22] Adewumi O. A., Singh V., Singh G. (2020). Chemical composition, traditional uses and biological activities of *Artemisia* species. *Journal of Pharmacognosy and Phytochemistry*.

[23] World Flora Online http://www.worldfloraonline.org/.

[24] Sanz M., Vilatersana R., Hidalgo O. (2008). Molecular phylogeny and evolution of floral characters of *Artemisia* and allies (Anthemideae, Asteraceae): evidence from nrDNA ETS and ITS sequences. *Taxon*.

[25] Abad M. J., Bedoya L. M., Apaza L., Bermejo P. (2012). The *artemisia* L. Genus: a review of bioactive essential oils. *Molecules*.

[26] Septembre-Malaterre A., Lalarizo Rakoto M., Marodon C. (2020). *Artemisia annua*, a traditional plant brought to light. *International Journal of Molecular Sciences*.

[27] Funk V. A., Bayer R. J., Keeley S. C. (2005). Everywhere but Antarctica: using a supertree to understand the diversity and distribution of the compositae. *Plant Diversity and Complexity Patterns: Local, Regional, and Global Dimensions*.

[28] Alesaeidi S., Miraj S. (2016). A systematic review of anti-malarial properties, immunosuppressive properties, anti-inflammatory properties, and anti-cancer properties of *Artemisia annua*. *Electron Physician*.

[29] Sanz M., Schneeweiss G. M., Vilatersana R., Vallès J. (2011). Temporal origins and diversification of *Artemisia* and allies (Anthemideae, Asteraceae). *Collectanea Botanica*.

[30] Valles J., McArthur E. D., Mcarthur E. D., Fairbanks D. J. (2001). *Artemisia* systematics and phylogeny: cytogenetic and molecular insights. *Shrubland ecosystem genetics and biodiversity: proceedings*.

[31] Valles J., Garnatje T., Sharma A. (2005). *Artemisia* and its allies: genome organization and evolution and their biosystematic, taxonomic and phylogenetic implications in the Artemisiinae and related subtribes (Asteraceae, Anthemideae). *Plant Genome: Biodiversity and Evolution*.

[32] Pellicer J., Garnatje T., Molero J., Pustahija F., Siljak-Yakovlev S., Valles J. (2010). Origin and evolution of the South American endemic *Artemisia* species (Asteraceae): evidence from molecular phylogeny, ribosomal DNA and genome size data. *Australian Journal of Botany*.

[33] Garcia S., McArthur E. D., Pellicer J., Sanderson S. C., Vallès J., Garnatje T. (2011). A molecular phylogenetic approach to western North America endemic *Artemisia* and allies (Asteraceae): untangling the sagebrushes. *American Journal of Botany*.

[34] Ling Y. R. (1991). The Old World Seriphidium (Compositae). *Bulletin of Botanical Research*.

[35] Ling Y. R. (1994). The genera *Artemisia* L. and *Seriphidium* (Bess.) Poljak. in the world. *Compositae Newslett*.

[36] De Clerck C., Genva M., Jijakli M. H., Fauconnier M.-L. (2021). Use of essential oils and volatile compounds as biological control agents. *Foods*.

[37] Başer K., Buchbauer G. (2010). Handbook of Essential Oils: Science. *Technology and Applications*.

[38] Kaul P. N., Bhattacharya A. K., Rajeswara Rao B. R., Syamasundar K. V., Ramesh S. (2003). Volatile constituents of essential oils isolated from different parts of cinnamon (*Cinnamomum zeylanicum Blume*). *Journal of the Science of Food and Agriculture*.

[39] Vieira T. M., Dias H. J., Medeiros T. C. (2017). Chemical composition and antimicrobial activity of the essential oil of *Artemisia absinthium* Asteraceae leaves. *Journal of Essential Oil Bearing Plants*.

[40] Liang J.-y., Wang W.-t., Zheng Y.-f. (2017). Bioactivities and chemical constituents of essential oil extracted from *Artemisia anethoides* against two stored product insects. *Journal of Oleo Science*.

[41] Liu H., Guo S. S., Lu L. (2021). Essential oil from *Artemisia annua* aerial parts: composition and repellent activity against two storage pests. *Natural Product Research*.

[42] Zhigzhitzhapova S. V., Dylenova E. P., Gulyaev S. M. (2020). Composition and antioxidant activity of the essential oil of *Artemisia annuaL*. *Natural Product Research*.

[43] Russo A., Bruno M., Avola R., Cardile V., Rigano D. (2020). Chamazulene-rich *Artemisia arborescens* essential oils affect the cell growth of human melanoma cells. *Plants*.

[44] Guan X., Ge D., Li S., Huang K., Liu J., Li F. (2019). Chemical composition and antimicrobial activities of *Artemisia argyi* Lévl. et Vant essential oils extracted by simultaneous distillation-extraction, subcritical extraction and hydrodistillation. *Molecules*.

[45] Rocha M. I., Gonçalves M. J., Cavaleiro C. (2021). Chemical characterization and bioactive potential of *Artemisia campestris* L. subsp. *maritima* (DC) Arcang. essential oil and hydrodistillation residual water. *Journal of Ethnopharmacology*.

[46] Ammar S., Noui H., Djamel S. (2020). Essential oils from three Algerian medicinal plants (*Artemisia campestris*, *Pulicaria arabica*, and *Saccocalyx satureioides*) as new botanical insecticides?. *Environmental Science and Pollution Research*.

[47] Al Jahid A., Elamrani A., Lahlou F. A. (2017). Chemical composition and antibacterial activity of the essential oil isolated from the seeds of Moroccan *Artemisia campestrisL*. *Journal of Essential Oil Bearing Plants*.

[48] Abidi A., Sebai E., Dhibi M. (2018). Chemical analyses and anthelmintic effects of *Artemisia campestris* essential oil. *Veterinary Parasitology*.

[49] Behbahani B. A., Shahidi F., Yazdi F. T., Mortazavi S. A., Mohebbi M. (2017). Antioxidant activity and antimicrobial effect of tarragon (*Artemisia dracunculus*) extract and chemical composition of its essential oil. *Journal of Food Measurement and Characterization*.

[50] Szczepanik M., Walczak M., Zawitowska B. (2018). Chemical composition, antimicromicrobial activity and insecticidal activity against the lesser mealworm *Alphitobius diaperinus* (Panzer)(Coleoptera: Tenebrionidae) of *Origanum vulgare* L. ssp. hirtum (Link) and Artemisia dracunculus L. essential oils. *Journal of the Science of Food and Agriculture*.

[51] Zhigzhitzhapova S. V., Namzalov B. T. B., Radnaeva L. D. (2021). Composition of Essential Oil of *Artemisia gmelinii* Web. ex Stechm. of Рriolkhonian Flora (Lake Baikal). *Ecology*.

[52] Xu Q., Zhang L., Yu S., Xia G., Zhu J., Zang H. (2021). Chemical composition and biological activities of an essential oil from the aerial parts of *Artemisia Gmelinii* weber ex Stechm. *Natural Product Research*.

[53] Qadir M., Maurya A. K., Waza A. A., Agnihotri V. K., Shah W. A. (2020). Chemical composition, antioxidant and cytotoxic activity ofArtemisia gmeliniiessential oil growing wild in Kashmir valley. *Natural Product Research*.

[54] Amor G., Caputo L., La Storia A., De Feo V., Mauriello G., Fechtali T. (2019). Chemical composition and antimicrobial activity of Artemisia herba-alba and *Origanum majorana* essential oils from Morocco. *Molecules*.

[55] Elmhalli F., Garboui S. S., Karlson A. K. B., Mozūraitis R., Baldauf S. L., Grandi G. (2021). Acaricidal activity against *Ixodes ricinus* nymphs of essential oils from the Libyan plants *Artemisia herba alba*, *Origanum majorana* and *Juniperus phoenicea*. *Veterinary Parasitology: Regional Studies and Reports*.

[56] Bellili S., Jazi S., Hrira M. Y. (2017). Phytochemical identification of volatile fraction, essential oil and screening of antioxidant, antibacterial, allelopathic and insecticidal potential from *Artemisia herba-alba* leaves. *Main Group Chemistry*.

[57] Jaradat N. (2021). Phytochemical profile and in vitro antioxidant, antimicrobial, vital physiological enzymes inhibitory and cytotoxic effects of *Artemisia jordanica* leaves essential oil from palestine. *Molecules*.

[58] Al-Qudah M. A., Onizat M. A., Alshamari A. K. (2021). Chemical composition and antioxidant activity of Jordanian *Artemisia judaica* L. as affected by different drying methods. *International Journal of Food Properties*.

[59] Guetat A., Al-Ghamdi F. A., Osman A. K. (2017). The genus *Artemisia* L. in the northern region of Saudi Arabia: essential oil variability and antibacterial activities. *Natural Product Research*.

[60] González S. B., Gastaldi B., Catalán C. (2019). Artemisia magellanica. Chemical composition of the essential oil from an unexplored endemic species of Patagonia. *Chemistry & Biodiversity*.

[61] Romeilah R. M., El-Beltagi H. S., Shalaby E. A. (2021). Antioxidant and cytotoxic activities of Artemisia monosperma L. and *Tamarix aphylla* L. essential oils. *Notulae Botanicae Horti Agrobotanici Cluj-Napoca*.

[62] Mishra T., Srivastava M., Kumar A., Pal M., Tewari S. (2017). Chemical composition and termiticidal activity of *Artemisia nilagirica* Essential oil growing in Southern Hilly regions of India. *Journal of Essential Oil Bearing Plants*.

[63] Sainz P., Andrés M. F., Martínez-Díaz R. A. (2019). Chemical composition and biological activities of *Artemisia pedemontana* subsp. assoana essential oils and hydrolate. *Biomolecules*.

[64] Dehghani Bidgoli R. (2021). Chemical composition of essential oil and antifungal activity of *Artemisia persica* Boiss. from Iran. *Journal of Food Science and Technology*.

[65] Jiang C.-Y., Zhou S.-X., Toshmatov Z. (2022). Chemical composition and phytotoxic activity of the essential oil of *Artemisia sieversiana* growing in Xinjiang, China. *Natural Product Research*.

[66] Qadir M., Maurya A. K., Agnihotri V. K., Shah W. A. (2021). Volatile composition, antibacterial and antioxidant activities of *Artemisia tourne fortiana* Reichb. from Kashmir, India. *Natural Product Research*.

[67] Malik S., de Mesquita L. S. S., Silva C. R. (2019). Chemical profile and biological activities of essential oil from Artemisia vulgaris L. cultivated in Brazil. *Pharmaceuticals*.

[68] Judžentienė A., Būdienė J. (2021). Mugwort (*Artemisia vulgaris* L.) essential oils rich in germacrene D, and their toxic activity. *Journal of Essential Oil Research*.

[69] Elansary H. O., Szopa A., Kubica P., Ekiert H., El-Ansary D. O., Al-Mana A. (2020). Polyphenol content and biological activities of Ruta graveolens L. and *Artemisia abrotanum* L. in Northern Saudi Arabia. *Processes*.

[70] Zhang X., Zhao Y., Guo L., Qiu Z., Huang L., Qu X. (2017). Differences in chemical constituents of *Artemisia annua* L from different geographical regions in China. *Plos ONE*.

[71] Al Jahid A., Essabaq S., Elamrani A., Blaghen M., Jamal Eddine J. (2016). Chemical composition, antimicrobial and antioxidant activities of the essential oil and the hydro-alcoholic extract of *Artemisia campestris* L. leaves from southeastern Morocco. *Journal of Biologically Active Products from Nature*.

[72] Yu F., Qian H., Zhang J., Sun J., Ma Z. (2018). Simultaneous quantification of eight organic acid components in *Artemisia capillaris* Thunb (Yinchen) extract using high-performance liquid chromatography coupled with diode array detection and high-resolution mass spectrometry. *Journal of Food and Drug Analysis*.

[73] Kiani B. H., Ullah N., Mirza B. (2019). Transgenic *Artemisia dubia* WALL showed altered phytochemistry and pharmacology. *Arabian Journal of Chemistry*.

[74] Lee Y. K., Hong E. Y., Whang W. K. (2017). Inhibitory effect of chemical constituents isolated from *Artemisia iwayomogi* on polyol pathway and simultaneous quantification of major bioactive compounds. *BioMed Research International*.

[75] Amkiss S., Dalouh A., Idaomar M. (2021). Chemical composition, genotoxicity and antigenotoxicity study of *Artemisia herba-alba* using the eye and wing SMART assay of *Drosophila melanogaster*. *Arabian Journal of Chemistry*.

[76] Zeng Y. T., Jiang J. M., Lao H. Y., Guo J. W., Lun Y. N., Yang M. (2015). Antitumor and apoptotic activities of the chemical constituents from the ethyl acetate extract of *Artemisia indica*. *Molecular Medicine Reports*.

[77] He Z.-Z., Yan J.-F., Song Z.-J. (2009). Chemical constituents from the aerial parts of *Artemisia minor*. *Journal of Natural Products*.

[78] Wang Q., Wang M. L., He X., Wang Q. (2021). Structural elucidation of two new diphenylethanes from *Artemisia mongolica*. *Chemistry of Natural Compounds*.

[79] Zan K., Chen X.-Q., Zhao M.-B., Tu P.-F. (2016). Sesquiterpenoids from aerial parts of *Artemisia myriantha*. *China Journal of Chinese Materia Medica*.

[80] Panasenko O., Mozul V., Denysenko O., Aksonova I., Oberemko T. (2021). *Characteristic of the Chemical Composition of Artemisia Pontica L*.

[81] Wu T., He F., Ma Q. L., Chen J., Aisa H. A. (2017). Chemical constituents of *Artemisia rupestris*. *Chemistry of Natural Compounds*.

[82] Stojanović G. S., Ickovski J. D., Đorđević A. S. (2020). The first report on chemical composition and antimicrobial activity of *Artemisia scoparia* Waldst. et Kit. extracts. *Natural Product Communications*.

[83] Geng C. A., Huang X. Y., Chen X. L. (2015). Three new anti-HBV active constituents from the traditional Chinese herb of Yin-Chen (*Artemisia scoparia*). *Journal of Ethnopharmacology*.

[84] Suzhang Z., Wei Y. (2016). Study on the chemical constituents of *Artemisia scoparia*. *Journal of Xinjiang Medical University*.

[85] Heshmati Afshar F., Zadehkamand M., Rezaei Z., Delazar A., Tarhriz V., Asgharian P. (2021). Chemical compositions, antimicrobial effects, and cytotoxicity of Asia minor wormwood (*Artemisia splendens* Willd) growing in Iran. *BMC Chemistry*.

[86] Mojarrab M., Saremi G., Emami S. A. (2017). Evaluation of antioxidant activity and identification of main compounds of various extracts of *Artemisia turanica* aerial parts. *Research Journal of Pharmacognosy*.

[87] Safari S., Taherkhani M. (2018). Extraction and Identification of flavon from *Artemisia turanica* Krasch the extractwhich has been collected from Esfarayen, Khorasan province. *Eco-phytochemical Journal of Medicinal Plants*.

[88] Van Nguyen Thien T., Tran L. T. K., Nhu N. T. T. (2018). A new eudesmane-type sesquiterpene from the leaves of Artemisia vulgaris. *Chemistry of Natural Compounds*.

[89] Ramazani A., Sardari S., Zakeri S., Vaziri B. (2010). In vitro antiplasmodial and phytochemical study of five *Artemisia* species from Iran and in vivo activity of two species. *Parasitology Research*.

[90] Bilia A. R., Lazari D., Messori L., Taglioli V., Temperini C., Vincieri F. F. (2002). Simple and rapid physico-chemical methods to examine action of antimalarial drugs with hemin:. *Life Sciences*.

[91] Liu K. C.-S. C., Yang S.-L., Roberts M., Elford B., Phillipson J. (1992). Antimalarial activity of *Artemisia annua* flavonoids from whole plants and cell cultures. *Plant Cell Reports*.

[92] Bilia A. R., Sannella A. R., Vincieri F. F. (2008). Antiplasmodial effects of a few selected natural flavonoids and their modulation of artemisinin activity. *Natural Product Communications*.

[93] Salehi B., Sharifi-Rad J., Capanoglu E. (2019). *Cucurbita* plants: from farm to industry. *Applied Sciences*.

[94] Salehi B., Shetty M. S., Kumar N. V. A. (2019). *Veronica* plants-drifting from farm to traditional healing, food application, and phytopharmacology. *Molecules*.

[95] Semwal P., Painuli S., Abu-Izneid T. (2022). Diosgenin: an updated pharmacological review and therapeutic perspectives. *Oxidative Medicine and Cellular Longevity*.

[96] Salehi B., Quispe C., Chamkhi I. (2021). Pharmacological properties of chalcones: a review of preclinical including molecular mechanisms and clinical evidence. *Frontiers in Pharmacology*.

[97] Scheau C., Caruntu C., Badarau I. A. (2021). Cannabinoids and inflammations of the gut-lung-skin barrier. *Journal of Personalized Medicine*.

[98] Georgiadis G., Zisis I. E., Docea A. O. (2020). Current concepts on the reno-protective effects of phosphodiesterase 5 inhibitors in acute kidney injury: systematic search and review. *Journal of Clinical Medicine*.

[99] Ali M., Abbasi B. H. (2013). Production of commercially important secondary metabolites and antioxidant activity in cell suspension cultures of *Artemisia absinthium* L. *Industrial Crops and Products*.

[100] Bora K. S., Sharma A. (2011). Evaluation of antioxidant and free-radical scavenging potential of *Artemisia absinthium*. *Pharmaceutical Biology*.

[101] Craciunescu O., Constantin D., Gaspar A., Toma L., Utoiu E., Moldovan L. J. C. C. J. (2012). Evaluation of antioxidant and cytoprotective activities of *Arnica montana* L. and *Artemisia absinthium* L. *Ethanolic Extracts*.

[102] Salehi B., Prakash Mishra A., Nigam M. (2021). *Ficus* plants: state of the art from a phytochemical, pharmacological, and toxicological perspective. *Phytotherapy Research*.

[103] Sharifi-Rad J., Dey A., Koirala N. (2021). *Cinnamomum* species: bridging phytochemistry knowledge, pharmacological properties and toxicological safety for health benefits. *Frontiers in Pharmacology*.

[104] Painuli S., Quispe C., Herrera-Bravo J. (2022). Nutraceutical profiling, bioactive composition, and biological applications of *Lepidium sativum* L. *Oxidative Medicine and Cellular Longevity*.

[105] Islam M. S., Quispe C., Hossain R. (2021). Neuropharmacological effects of quercetin: a literature-based review. *Frontiers in Pharmacology*.

[106] Hadi A., Hossein N., Shirin P., Najmeh N., Abolfazl M. J. I. J. P. S. R. R. (2014). Anti-inflammatory and analgesic activities of *Artemisia absinthium* and chemical composition of its essential oil. *International Journal of Pharmaceutical Sciences Review and Research*.

[107] Sharifi-Rad J., Quispe C., Patra J. K. (2021). Paclitaxel: application in modern oncology and nanomedicine-based cancer therapy. *Oxidative Medicine and Cellular Longevity*.

[108] Shafi G., Hasan T. N., Syed N. A. (2012). *Artemisia absinthium* (AA): a novel potential complementary and alternative medicine for breast cancer. *Molecular Biology Reports*.

[109] Sharifi-Rad J., Bahukhandi A., Dhyani P. (2021). Therapeutic potential of neoechinulins and their derivatives: an overview of the molecular mechanisms behind pharmacological activities. *Frontiers in Nutrition*.

[110] Docea A. O., Mitrut P., Grigore D., Pirici D., Calina D. C., Gofita E. (2012). Immunohistochemical expression of TGF beta (TGF-*β*), TGF beta receptor 1 (TGFBR1), and Ki67 in intestinal variant of gastric adenocarcinomas. *Romanian Journal of Morphology and Embryology*.

[111] Dhyani P., Quispe C., Sharma E. (2022). Anticancer potential of alkaloids: a key emphasis to colchicine, vinblastine, vincristine, vindesine, vinorelbine and vincamine. *Cancer Cell International*.

[112] Amin R., Quispe C., Docea A. O. (2022). The role of tumour necrosis factor in neuroinflammation associated with Parkinson’s disease and targeted therapies. *Neurochemistry International*.

[113] Calina D., Buga A. M., Mitroi M. (2020). The treatment of cognitive, behavioural and motor impairments from brain injury and neurodegenerative diseases through cannabinoid system modulation-evidence from in vivo studies. *Journal of Clinical Medicine*.

[114] Buga A. M., Docea A. O., Albu C. (2019). Molecular and cellular stratagem of brain metastases associated with melanoma. *Oncology Letters*.

[115] Salehi B., Sestito S., Rapposelli S. (2019). Epibatidine: a promising natural alkaloid in health. *Biomolecules*.

[116] Zeng K.-W., Liao L.-X., Song X.-M. (2015). Caruifolin D from *Artemisia absinthium* L. inhibits neuroinflammation via reactive oxygen species-dependent c-jun N-terminal kinase and protein kinase c/NF-*κ*B signaling pathways. *European Journal of Pharmacology*.

[117] Mohammadian A., Moradkhani S., Ataei S. (2016). Antioxidative and hepatoprotective effects of hydroalcoholic extract of *Artemisia absinthium* L. in rat. *Journal of Herbmed Pharmacology*.

[118] Sharifi-Rad J., Quispe C., Herrera-Bravo J. (2021). Phytochemical constituents, biological activities, and health-promoting effects of the *Melissa officinalis*. *Oxidative Medicine and Cellular Longevity*.

[119] Hossain R., Quispe C., Herrera-Bravo J. (2021). *Lasia spinosa* chemical composition and therapeutic potential: a literature-based review. *Oxidative Medicine and Cellular Longevity*.

[120] Kartikadewi A., Prasetyo A., Budipradigdo L., Nugroho H., Tjahjono K., Lelono A. J. T. I. B. J. (2019). *Artemisia annua* leaf extract increases GLUT-4 expression in type 2 diabetes mellitus rat. *The Indonesian Biomedical Journal*.

[121] Daradka H. M., Abas M. M., Mohammad M. A., Jaffar M. M. J. C. C. P. (2014). Antidiabetic effect of *Artemisia absinthium* extracts on alloxan-induced diabetic rats. *Comparative Clinical Pathology*.

[122] Wang J., Xu C., Wong Y. K. (2019). Artemisinin, the magic drug discovered from traditional Chinese medicine. *Engineering*.

[123] Suresh J., Mahesh N., Ahuja J., Santilna K. (2011). Review on *Artemisia nilagirica* (Clarke) pamp. *Journal of Biologically Active Products from Nature*.

[124] Oliveira F. D., Andrade L. N., De Sousa É. B., De Sousa D. P. (2014). Anti-ulcer activity of essential oil constituents. *Molecules*.

[125] Devmurari V., Jivani N. (2010). Anticancer Evaluation of *Artemisia Nilagirica*. *International Journal of Pharmtech Research*.

[126] Gordanian B., Behbahani M., Carapetian J., Fazilati M. (2014). In vitro evaluation of cytotoxic activity of flower, leaf, stem and root extracts of five *Artemisia* species. *Research in Pharmaceutical Sciences*.

[127] Sahu N., Meena S., Shukla V. (2018). Extraction, fractionation and re-fractionation of *Artemisia nilagirica* for anticancer activity and HPLC-ESI-QTOF-MS/MS determination. *Journal of Ethnopharmacology*.

[128] Ahamad J., Mir S., Amin S. (2019). A pharmacognostic review on *Artemisia absinthium*. *International Research Journal of Pharmacy*.

[129] Mojarrab M., Lagzian M.-S., Emami S. A., Asili J., Tayarani-Najaran Z. (2013). *In vitro* anti-proliferative and apoptotic activity of different fractions of *Artemisia armeniaca*. *Revista Brasileira de Farmacognosia*.

[130] Wang Z. Q., Ribnicky D., Zhang X. H. (2011). An extract of *Artemisia dracunculus* L. enhances insulin receptor signaling and modulates gene expression in skeletal muscle in KK- A^y^ mice. *The Journal of Nutritional Biochemistry*.

[131] Ribnicky D. M., Kuhn P., Poulev A. (2009). Improved absorption and bioactivity of active compounds from an anti-diabetic extract of *Artemisia dracunculus* L. *International Journal of Pharmaceutics*.

[132] Mansi K., Lahham J. (2008). Effects of *Artemisia sieberi* Besser (a. herba-alba) on heart rate and some hematological values in normal and alloxan-induced diabetic rats. *Journal of Basic and Applied Sciences*.

[133] Esmaeili F., Sepehri G., Moshtaghi-Kashanian G.-R., Khaksari M., Salari N., Sepehri E. (2009). The effect of acute administration of *Artemisia Persia* extracts on arterial blood pressure and heart rate in rats. *American Journal of Applied Sciences*.

[134] Amat N., Upur H., Blažeković B. (2010). In vivo hepatoprotective activity of the aqueous extract of *Artemisia absinthium* L. against chemically and immunologically induced liver injuries in mice. *Journal of Ethnopharmacology*.

[135] Ahameethunisa A. R., Hopper W. (2010). Antibacterial activity of *Artemisia nilagirica* leaf extracts against clinical and phytopathogenic bacteria. *BMC Complementary and Alternative Medicine*.

[136] Amin S. M., Hassan H. M., El Gendy A. E. N. G. (2019). Comparative chemical study and antimicrobial activity of essential oils of three *Artemisia* species from Egypt and Saudi Arabia. *Flavour and Fragrance Journal*.

[137] Mokhtar A. B., Ahmed S. A., Eltamany E. E., Karanis P. (2019). Anti-blastocystis activity *in vitro* of Egyptian herbal extracts (Family: Asteraceae) with emphasis on Artemisia judaica. *International Journal of Environmental Research and Public Health*.

[138] Sonker N., Pandey A. K., Singh P. (2015). Efficiency of *Artemisia nilagirica* (Clarke) Pamp. essential oil as a mycotoxicant against postharvest mycobiota of table grapes. *Journal of the Science of Food and Agriculture*.

[139] Perazzo F. F., Lima L. M., Maistro E. L., Carvalho J. E., Rehder V. L., Carvalho J. C. (2008). Effect of *Artemisia annua* L. leaves essential oil and ethanol extract on behavioral assays. *Farmacognosia*.

[140] Mahmoudi M., Ebrahimzadeh M., Ansaroudi F., Nabavi S., Nabavi S. (2009). Antidepressant and antioxidant activities of *Artemisia absinthium* L. at flowering stage. *African Journal of Biotechnology*.

[141] Rahman M., Ali M., Sharif M., Tajmim A. (2017). A review study on the traditional plants has potential antidepressant property. *MOJ Cell Science & Report*.

[142] Woo T.-S., Yoon S.-Y., Pena I. C. D. (2011). Anticonvulsant effect of *Artemisia capillaris* Herba in mice. *Biomolecules & Therapeutics*.

[143] Pal P., Ghosh A. (2018). Antioxidant, anti-Alzheimer and anti-Parkinson activity of *Artemisia nilagirica* leaves with flowering tops. *Pharmaceutical and Biosciences Journal*.

[144] Skowyra M., Gallego M. G., Segovia F., Almajano M. P. (2014). Antioxidant properties of *Artemisia annua* extracts in model food emulsions. *Antioxidants*.

[145] Afshar F. H., Delazar A., Janneh O. (2011). Evaluation of antimalarial, free-radical-scavenging and insecticidal activities of *Artemisia scoparia* and *A. spicigera*, Asteraceae. *Revista Brasileira De Farmacognosia*.

[146] Leeja L., Thoppil E. J. (2004). Essential oil composition and mosquito larvicidal activity of *Artemisia nilagirica* (CB Clarke) Pamp. from South India. *Journal of Phytological Research*.

[147] Prashant R. V., Subburaju R., Balakrishnan N. (2006). Larvicidal activity of *Artemisia nilagirica* (Clarke) Pamp. and *Ocimum sanctum* L. A preliminary study. *Journal of Natural Remedies*.

[148] Faizei F., Maghsood A. H., Parandin F., Matini M., Moradkhani S., Fallah M. (2015). Antiprotoscolices effect of methanolic extract of *Zingiber officinale*, *Artemisia aucheri* and *Eucalyp-tus globulus* against Echinococcus granulosus in vitro. *Iranian Journal of Pharmacology and Therapeutics*.

[149] Ryu K., Yoou M., Seo Y., Yoon K., Kim H., Jeong H. (2018). Therapeutic effects of *Artemisia scoparia* Waldst. et Kitaib in a murine model of atopic dermatitis. *Clinical and Experimental Dermatology*.

[150] Sajid M., Khan M. R., Shah N. A. (2016). Proficiencies of *Artemisia scoparia* against CCl 4 induced DNA damages and renal toxicity in rat. *BMC Complementary and Alternative Medicine*.

[151] Ha H., Lee H., Seo C. S. (2014). *Artemisia capillaris* inhibits atopic dermatitis-like skin lesions in Dermatophagoides farinae-sensitized Nc/Nga mice. *BMC Complementary and Alternative Medicine*.

[152] Honmore V., Kandhare A., Zanwar A. A., Rojatkar S., Bodhankar S., Natu A. (2015). *Artemisia pallens*alleviates acetaminophen induced toxicity via modulation of endogenous biomarkers. *Pharmaceutical Biology*.

[153] Zime-Diawara H., Ganfon H., Gbaguidi F. (2015). The antimalarial action of aqueous and hydro alcoholic extracts of *Artemisia annua* L. cultivated in Benin: *In vitro* and *in vivo* studies. *Journal of Chemical and Pharmaceutical Research*.

[154] Stebbings S., Beattie E., McNamara D., Hunt S. (2016). A pilot randomized, placebo-controlled clinical trial to investigate the efficacy and safety of an extract of *Artemisia annua* administered over 12 weeks, for managing pain, stiffness, and functional limitation associated with osteoarthritis of the hip and knee. *Clinical Rheumatology*.

[155] Hunt S., Stebbings S., McNamara D. (2016). An open-label six-month extension study to investigate the safety and efficacy of an extract of *Artemisia annua* for managing pain, stiffness and functional limitation associated with osteoarthritis of the hip and knee. *The New Zealand Medical Journal*.

[156] Basiri Z., Zeraati F., Esna-Ashari F. (2017). Topical effects of *Artemisia Absinthium* ointment and liniment in comparison with piroxicam gel in patients with knee joint osteoarthritis: a randomized double-blind controlled trial. *Iranian Journal of Medical Sciences*.

[157] Krebs S., Omer T. N., Omer B. (2010). Wormwood (*Artemisia absinthium*) suppresses tumour necrosis factor alpha and accelerates healing in patients with Crohn's disease - A controlled clinical trial. *Phytomedicine*.

[158] Remberg P., Björk L., Hedner T., Sterner O. (2004). Characteristics, clinical effect profile and tolerability of a nasal spray preparation of *Artemisia abrotanum* L. for allergic rhinitis. *Phytomedicine*.

[159] Yu J., Wang G., Jiang N. (2020). Study on the Repairing Effect of Cosmetics Containing *Artemisia* annua on Sensitive Skin. *Journal of Cosmetics, Dermatological Sciences and Applications*.

[160] Konstat-Korzenny E., Ascencio-Aragón J. A., Niezen-Lugo S., Vázquez-López R. (2018). Artemisinin and its synthetic derivatives as a possible therapy for cancer. *Medical Sciences*.

[161] Slezáková S., Ruda-Kucerova J. (2017). Anticancer activity of artemisinin and its derivatives. *Anticancer Research*.

[162] Dell’Eva R., Pfeffer U., Vené R. (2004). Inhibition of angiogenesis in vivo and growth of Kaposi's sarcoma xenograft tumors by the anti-malarial artesunate. *Biochemical Pharmacology*.

[163] Zhang Y., Xu G., Zhang S., Wang D., Prabha P. S., Zuo Z. (2018). Antitumor research on artemisinin and its bioactive derivatives. *Natural Products and Bioprospecting*.

[164] Crespo-Ortiz M. P., Wei M. Q. (2012). Antitumor activity of artemisinin and its derivatives: from a well-known antimalarial agent to a potential anticancer drug. *Journal of Biomedicine and Biotechnology*.

[165] Jansen F. H., Adoubi I., de Cnodder T., Jansen N., Tschulakow A., Efferth T. (2011). First study of oral Artenimol-R in advanced cervical cancer: clinical benefit, tolerability and tumor markers. *Anticancer Research*.

[166] Zhang Z.-Y., Yu S.-Q., Miao L.-Y. (2008). Artesunate combined with vinorelbine plus cisplatin in treatment of advanced non-small cell lung cancer: a randomized controlled trial. *Journal of Chinese Integrative Medicine*.

[167] Singh N., Verma K. (2002). Case report of a laryngeal squamous cell carcinoma treated with artesunate. *Archive of Oncology*.

[168] Berger T. G., Dieckmann D., Efferth T. (2005). Artesunate in the treatment of metastatic uveal melanoma-first experiences. *Oncology Reports*.

[169] Singh N. P., Panwar V. K. (2006). Case report of a pituitary macroadenoma treated with artemether. *Integrative Cancer Therapies*.

[170] Mendez-del Villar M., Puebla-Pérez A. M., Sanchez-Pena M. J., González-Ortiz L. J., Martinez-Abundis E., González-Ortiz M. (2016). Effect of *Artemisia dracunculus* administration on glycemic control, insulin sensitivity, and insulin secretion in patients with impaired glucose tolerance. *Journal of Medicinal Food*.

[171] Choi J.-Y., Shin S.-K., Jeon S.-M. (2011). Dose–response study of sajabalssuk ethanol extract from *Artemisia princeps* Pampanini on blood glucose in subjects with impaired fasting glucose or mild type 2 diabetes. *Journal of Medicinal Food*.

[172] Li Y., Zheng M., Zhai X. (2015). Effect OF-GYMNEMA sylvestre, Citrullus colocynthis and *Artemisia absinthium* on blood glucose and lipid profile in diabetic human. *Acta Poloniae Pharmaceutica*.

[173] Mohammadi S., Jafari B., Asgharian P., Martorell M., Sharifi-Rad J. (2020). Medicinal plants used in the treatment of Malaria: a key emphasis to *Artemisia, Cinchona*, Cryptolepis, and *Tabebuia genera*. *Phytotherapy Research*.

[174] Martínez M. J. A., Del Olmo L. M. B., Ticona L. A. (2012). Genus: a review of bioactive sesquiterpene lactones. *Studies in Natural Products Chemistry*.

[175] Li Y. (2012). Qinghaosu (artemisinin): chemistry and pharmacology. *Acta Pharmacologica Sinica*.

[176] Warsame M., Gyapong M., Mpeka B. (2016). Pre-referral rectal artesunate treatment by community-based treatment providers in Ghana, Guinea-Bissau, Tanzania, and Uganda (study 18): a cluster-randomized trial. *Clinical Infectious Diseases*.

[177] Dondorp A. M., Fanello C. I., Hendriksen I. C. (2010). Artesunate versus quinine in the treatment of severe falciparum malaria in African children (AQUAMAT): an open-label, randomised trial. *The Lancet*.

[178] Group, A (2001). A meta-analysis using individual patient data of trials comparing artemether with quinine in the treatment of severe falciparum malaria. *Transactions of the Royal Society of Tropical Medicine and Hygiene*.

[179] Dobaño C., Nhabomba A. J., Manaca M. N. (2019). A balanced Proinflammatory and regulatory cytokine signature in young African children is associated with lower risk of clinical malaria. *Clinical Infectious Diseases*.

[180] Das S., Manna S., Saha B., Hati A. K., Roy S. (2019). Novel pfkelch13 gene polymorphism associates with artemisinin resistance in eastern India. *Clinical Infectious Diseases*.

[181] Eastman R. T., Fidock D. A. (2009). Artemisinin-based combination therapies: a vital tool in efforts to eliminate malaria. *Nature Reviews Microbiology*.

[182] Pull L., Lupoglazoff J.-M., Beardmore M. (2019). Artenimol–piperaquine in children with uncomplicated imported falciparum malaria: experience from a prospective cohort. *Malaria Journal*.

[183] Leblanc C., Vasse C., Minodier P. (2020). Prise en charge et prevention du paludisme d'importation de l'enfant. Mise a jour des recommandations pour la pratique clinique 2007. *Medecine et Maladies Infectieuses*.

[184] Ballard S.-B., Salinger A., Desai M., Tan K. R. (2018). Updated CDC recommendations for using artemether-lumefantrine for the treatment of uncomplicated malaria in pregnant women in the United States. *Morbidity and Mortality Weekly Report*.

[185] Daddy N. B., Kalisya L. M., Bagire P. G., Watt R. L., Towler M. J., Weathers P. J. (2017). *Artemisia annua* dried leaf tablets treated malaria resistant to ACT and i.v. artesunate: Case reports. *Phytomedicine*.

[186] Munyangi J., Cornet-Vernet L., Idumbo M. (2019). RETRACTED: *Artemisia annua* and *Artemisia afra* tea infusions *vs*. artesunate-amodiaquine (ASAQ) in treating *Plasmodium falciparum* malaria in a large scale, double blind, randomized clinical trial. *Phytomedicine*.

[187] Willcox M. L., Burton S., Oyweka R., Namyalo R., Challand S., Lindsey K. (2011). Evaluation and pharmacovigilance of projects promoting cultivation and local use of *Artemisia annua* for malaria. *Malaria Journal*.

[188] Calina D., Sarkar C., Arsene A. L. (2020). Recent advances, approaches and challenges in targeting pathways for potential COVID-19 vaccines development. *Immunologic Research*.

[189] Calina D., Hartung T., Docea A. O. (2020). COVID-19 vaccines: ethical framework concerning human challenge studies. *DARU Journal of Pharmaceutical Sciences*.

[190] Sohrabi C., Alsafi Z., O'Neill N. (2020). World Health Organization declares global emergency: a review of the 2019 novel coronavirus (COVID-19). *International Journal of Surgery*.

[191] Zhuang W., Fan Z., Chu Y. (2020). Chinese patent medicines in the treatment of coronavirus disease 2019 (COVID-19) in China. *Frontiers in Pharmacology*.

[192] World Health Organization SARS: Clinical Trials on Treatment Using a Combination of Traditional Chinese Medicine and Western Medicine.

[193] Zhao Z., Li Y., Zhou L. (2021). Prevention and treatment of COVID-19 using traditional Chinese medicine: a review. *Phytomedicine*.

[194] Shapira M. Y., Resnick I. B., Chou S. (2008). Artesunate as a potent antiviral agent in a patient with late drug-resistant cytomegalovirus infection after hematopoietic stem cell transplantation. *Clinical Infectious Diseases*.

[195] Lachenmeier D. W., Uebelacker M. (2010). Risk assessment of thujone in foods and medicines containing sage and wormwood - Evidence for a need of regulatory changes?. *Regulatory Toxicology and Pharmacology*.

[196] Lachenmeier D. W. (2010). Wormwood (*Artemisia absinthium* L.) --A curious plant with both neurotoxic and neuroprotective properties?. *Journal of Ethnopharmacology*.

[197] Nofal S. M., Mahmoud S. S., Ramadan A., Soliman G., Fawzy R. (2009). Anti-diabetic effect of *Artemisia judaica* extracts. *Research Journal of Medicine and Medical Sciences*.

[198] Paramakrishnan N., Ahuja J., Suresh J., Khan M., Sebastian M. (2012). Evaluation of acute oral toxicity of aerial parts of *Artemisia parviflora* Roxb. *Der Pharmacia Sinica*.

[199] Judzentiene A., Garjonyte R. (2016). Compositional variability and toxic activity of Mugwort (*Artemisia vulgaris*) essential oils. *Natural Product Communications*.

[200] Siddiqui M., Waghmare S., Hajare S., Deshmukh R. I. S., Ali S. C. S. A. (2018). Phytochemical analysis and acute toxicity studies of *Artemisia annua* in Swiss albino mice. *Journal of Pharmacognosy and Phytochemistry*.

[201] Classen W., Altmann B., Gretener P., Souppart C., Skelton-Stroud P., Krinke G. (1999). Differential effects of orally versus parenterally administered qinghaosu derivative artemether in dogs. *Experimental and Toxicologic pathology*.

[202] Ogbole E., Isaiah I., Ogundeko T., Asalu A., Modupe B., Aguiyi J. (2014). Acute toxicity studies of locally cultivated Artemisia annua leaf extract in Rats. *World Journal of Pharmaceutical Sciences*.

[203] Qureshi S., Ageel A., Al-Yahya M., Tariq M., Mossa J., Shah A. (1990). Preliminary toxicity studies on ethanol extracts of the aerial parts of *Artemisia abyssinica* and *A. inculta* in mice. *Journal of Ethnopharmacology*.

[204] Mukinda J. T., Syce J. A. (2007). Acute and chronic toxicity of the aqueous extract of *Artemisia afra* in rodents. *Journal of Ethnopharmacology*.

[205] Nontprasert A., Nosten-Bertrand M., Pukrittayakamee S., Vanijanonta S., Angus B. J., White N. J. (1998). Assessment of the neurotoxicity of parenteral artemisinin derivatives in mice. *The American Journal of Tropical Medicine and Hygiene*.

[206] Trendafilova A., Moujir L. M., Sousa P. M. C., Seca A. M. L. (2021). Research advances on health effects of edible *Artemisia* species and some sesquiterpene lactones constituents. *Foods*.

[207] Padosch S. A., Lachenmeier D. W., Kröner L. U. (2006). Absinthism: a fictitious 19th century syndrome with present impact. *Substance Abuse Treatment, Prevention, and Policy*.

[208] Lachenmeier D. W., Walch S. G., Padosch S. A., Kröner L. U. (2006). Absinthe—a review. *Critical Reviews in Food Science and Nutrition*.

[209] Laadraoui J., Aboufatima R., El Gabbas Z. (2018). Effect of *Artemisia herba-alba* consumption during pregnancy on fertility, morphological and behaviors of mice offspring. *Journal of Ethnopharmacology*.

[210] Oliaee D., Boroushaki M. T., Oliaee N., Ghorbani A. (2014). Evaluation of cytotoxicity and antifertility effect of *Artemisia kopet daghensis*. *Advances in Pharmacological Sciences*.

[211] Paulsen E. (2017). Systemic allergic dermatitis caused by sesquiterpene lactones. *Contact Dermatitis*.

[212] Wu P., He Y., Zeng Z., Yang Z., Li Y. (2020). Allergic contact dermatitis by *Artemisia*: report of two cases. *Contact Dermatitis*.

[213] Pablos I., Egger M., Vejvar E. (2019). Similar allergenicity to different *Artemisia* species is a consequence of highly cross-reactive Art v 1-like molecules. *Medicina*.

[214] Denisow-Pietrzyk M., Pietrzyk Ł., Denisow B. (2019). Asteraceae species as potential environmental factors of allergy. *Environmental Science and Pollution Research International*.

[215] Tang R., Sun J. L., Yin J., Li Z. (2015). *Artemisia* allergy research in China. *BioMed Research International*.

[216] Gao Z., Fu W.-Y., Sun Y. (2019). *Artemisia* pollen allergy in China: component-resolved diagnosis reveals allergic asthma patients have significant multiple allergen sensitization. *Allergy*.

[217] Hirschwehr R., Heppner C., Spitzauer S. (1998). Identification of common allergenic structures in mugwort and ragweed pollen. *Journal of Allergy and Clinical Immunology*.

[218] Zhou C., Gao Z.-S., Zheng M., Gilissen L. J. W. J., Shen H.-H., Frewer L. J. (2012). Mechanism of type I hypersensitivity. *Multidisciplinary Approaches to Allergies*.

[219] Oteros J., Bartusel E., Alessandrini F. (2019). *Artemisia* pollen is the main vector for airborne endotoxin. *Journal of Allergy and Clinical Immunology*.

[220] Brandys J., Grimsøen A., Nilsen B. M., Paulsen B. S., Park H. S., Hong C. S. (1993). Cross-reactivity between pollen extracts from six *Artemisia* species. *Planta Medica*.

[221] Efferth T., Kaina B. (2010). Toxicity of the antimalarial artemisinin and its dervatives. *Critical Reviews in Toxicology*.

[222] Xia M., Liu D., Liu Y., Liu H. (2020). The therapeutic effect of Artemisinin and its derivatives in kidney disease. *Frontiers in Pharmacology*.

[223] Dai X., Zhang X., Chen W. (2021). Dihydroartemisinin: a potential natural anticancer drug. *International Journal of Biological Sciences*.

[224] Anh C. X., Chavchich M., Birrell G. W. (2020). Pharmacokinetics and ex vivo antimalarial activity of artesunate-amodiaquine plus methylene blue in healthy volunteers. *Antimicrobial Agents and Chemotherapy*.

[225] Fu C., Shi H., Chen H., Zhang K., Wang M., Qiu F. (2021). Oral bioavailability comparison of artemisinin, deoxyartemisinin, and 10-deoxoartemisinin based on computer simulations and pharmacokinetics in rats. *ACS Omega*.

[226] Li X., Hu J., Yuan Y. (2021). Pharmacokinetics and toxicokinetics of artemisinin-hydroxychloroquine sulfate tablets in rats and dogs. *Evidence-Based Complementary and Alternative Medicine : eCAM*.

[227] Mancuso R. I., Foglio M. A., Olalla Saad S. T. (2021). Artemisinin-type drugs for the treatment of hematological malignancies. *Cancer Chemotherapy and Pharmacology*.

[228] Kitic D., Miladinovic B., Randjelovic M. (2022). Anticancer potential and other pharmacological properties of *Prunus armeniaca* L.: an updated overview. *Plants*.

[229] Tsoukalas D., Zlatian O., Mitroi M. (2021). A novel nutraceutical formulation can improve motor activity and decrease the stress level in a murine model of middle-age animals. *Journal of Clinical Medicine*.

[230] Alshehri M. M., Quispe C., Herrera-Bravo J. (2022). A review of recent studies on the antioxidant and anti-infectious properties of *senna* plants. *Oxidative Medicine and Cellular Longevity*.

[231] Quispe C., Herrera-Bravo J., Khan K. (2022). Therapeutic applications of curcumin nanomedicine formulations in cystic fibrosis. *Progress in Biomaterials*.

[232] Docea A. O., Calina D., Buga A. M. (2020). The effect of silver nanoparticles on antioxidant/pro-oxidant balance in a murine model. *International Journal of Molecular Sciences*.

